# *Cntnap4* partial deficiency exacerbates α-synuclein pathology through astrocyte–microglia C3-C3aR pathway

**DOI:** 10.1038/s41419-023-05807-y

**Published:** 2023-04-22

**Authors:** Wenlong Zhang, Liuyan Ding, Huaqing Chen, Mengran Zhang, Runfang Ma, Shaohui Zheng, Junwei Gong, Zhiling Zhang, Huaxi Xu, Pingyi Xu, Yunlong Zhang

**Affiliations:** 1 Department of Neurology, The First Affiliated Hospital of Guangzhou Medical University, Guangzhou, 510120 China; 2 Key Laboratory of Neurological Function and Health, School of Basic Medical Sciences, Guangzhou Medical University, Guangzhou, 511436 China; 3 Shenzhen Key Laboratory of Gene and Antibody Therapy, Center for Biotechnology and Biomedicine, State Key Laboratory of Chemical Oncogenomics, State Key Laboratory of Health Sciences and Technology, Institute of Biopharmaceutical and Health Engineering, Shenzhen International Graduate School, Tsinghua University, Shenzhen, Guangdong 518055 China; 4 School of Life Sciences, Westlake Laboratory of Life Sciences and Biomedicine, Westlake University, Hangzhou, 310024 China; 5 Institute for Brain Science and Disease, Chongqing Medical University, Chongqing, 400016 China

**Keywords:** Parkinson's disease, Microglia

## Abstract

Parkinson’s disease (PD) is the most common progressive neurodegenerative movement disorder, which is characterized by dopaminergic (DA) neuron death and the aggregation of neurotoxic α-synuclein. *Cntnap4*, a risk gene of autism, has been implicated to participate in PD pathogenesis. Here we showed *Cntnap4* lacking exacerbates α-synuclein pathology, nigrostriatal DA neuron degeneration and motor impairment, induced by injection of adeno-associated viral vector (AAV)-mediated human α-synuclein overexpression (AAV-*h*α-Syn). This scenario was further validated in A53T α-synuclein transgenic mice injected with AAV-Cntnap4 shRNA. Mechanistically, α-synuclein derived from damaged DA neuron stimulates astrocytes to release complement C3, activating microglial C3a receptor (C3aR), which in turn triggers microglia to secrete complement C1q and pro-inflammatory cytokines. Thus, the astrocyte–microglia crosstalk further drives DA neuron death and motor dysfunction in PD. Furthermore, we showed that in vivo depletion of microglia and microglial targeted delivery of a novel C3aR antagonist (SB290157) rescue the aggravated α-synuclein pathology resulting from *Cntnap4* lacking. Together, our results indicate that Cntnap4 plays a key role in α-synuclein pathogenesis by regulating glial crosstalk and may be a potential target for PD treatment.

## Introduction

The α-synuclein (encoded by *SNCA*) is a 14-kDa intracellular protein enriched in the presynaptic terminals, where it binds to lipids and modulates the release of synaptic vesicles. Genetically, duplications, triplications, and N-terminal point mutations of *SNCA* (A30P, E46K, H50Q, G51D, A53E, and A53T) cause autosomal dominant forms of familial Parkinson’s disease (PD) [1–7]. The molecular mechanisms underlying neuronal death in PD due to α-synuclein involve multiple pathways, such as dysfunctional synaptic-vesicle trafficking, mitochondrial dysfunction, oxidative stress, altered calcium homeostasis, defective autophagic degradation, impaired organelle dynamics, and neuroinflammation [8–12].

Over the last few decades, increasing evidence has suggested that astrocytes and microglia contribute to the α-synuclein pathology in PD [13]. Although most cytoplasmic α-synuclein inclusions are found in neurons, immunoreactive α-synuclein is also found in a subset of glia (astrocytes and oligodendrocytes) in the midbrain and basal ganglia of PD patients [14]. Mechanistically, loss of function of lysosomal *ATP13A2* in astrocytes and impaired tunneling nanotubes (TNTs) between astrocytes contribute to α-synuclein accumulation and propagation in PD [15]. Astrocytic dynamin-dependent endocytosis promotes the efficient uptake of α-synuclein fibrils [16]. Emerging evidence indicates that microglia also play a critical role in α-synuclein pathology in PD. Active microglia are closely associated with α-synuclein-positive deposits in the olfactory bulb, substantia nigra (SN), and pons of PD patients [17, 18]. Microglia-expressed lymphocyte-activation gene 3 (LAG3), which is genetically linked with PD, is a receptor for misfolded α-synuclein fibrils. Moreover, LAG3 participates in the α-synuclein spread between cells [19–21]. Microglia remove neuron-released α-synuclein via TLR4-NF-κB-p62-mediated selective autophagy [22], and also cooperatively degrade α-synuclein fibrils via TNTs [23]. However, the communication between astrocytes and microglia in α-synuclein pathology remains poorly understood.

Previously, we reported that loss of function of contactin-associated protein-like 4 (Cntnap4) induces parkinsonian phenotypes, such as dopaminergic (DA) neuronal death and movement disorders, by regulating mitophagy [24]. Herein, we report that *Cntnap4* partial deficiency accelerates α-synuclein pathology, nigrostriatal neuron degeneration, and motor disorders induced by the injection of adeno-associated viral vector (AAV)-mediating human α-synuclein overexpression (AAV-*h*α-Syn). Mechanistically, we found that *Cntnap4* partial deficiency exacerbates α-synuclein pathology and Cntnap4 is involved in modulating the interplay between astrocytes and microglia though the Complement 3 (C3)-C3a receptor (C3aR) signaling pathway. We also delineated this scenario in A53T α-synuclein transgenic mice injected with AAV-Cntnap4 shRNA. In vivo depletion of microglia by PLX3397 and microglial targeted delivery of a novel C3aR antagonist, SB290157, reduce *Cntnap4* partial deficiency-aggravated α-synuclein pathology. Hence, our study reveals a novel role of *Cntnap4* deficiency in PD pathogenesis through astrocyte–microglia crosstalk.

## Results

### *Cntnap4* partial deficiency exacerbates α-synuclein pathology, nigral DA neuronal death, and motor dysfunction in mice injected with AAV-*h*α-Syn

**Fig. 1:**
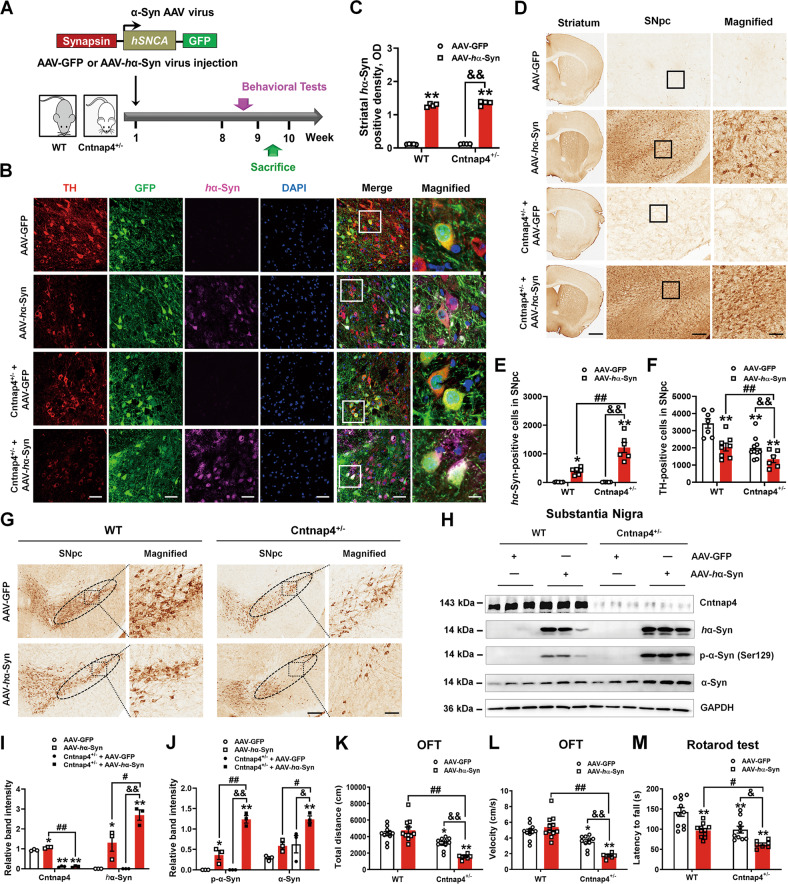
*Cntnap4* partial deficiency exacerbates α-synuclein pathology, nigral DA neuronal death, and motor dysfunction. **A** Schematic model of the study design. **B** Immunofluorescence staining of TH and *h*α-Syn in the SNpc of WT and Cntnap4^+/−^ mice injected with either AAV-GFP or AAV-*h*α-Syn. Scale bars: 40 μm. Magnified co-staining of TH and *h*α-Syn-positive cells in the SNpc are shown in the right column of panel **B**. Scale bars: 8 μm. **C**–**E** Immunohistochemical staining and quantification of *h*α-Syn density and positive cells in the striatum and SNpc of WT and Cntnap4^+/−^ mice injected with either AAV-GFP or AAV-*h*α-Syn. Scale bars: 1 mm in the striatum and 100 μm in the SNpc. Magnified images of *h*α-Syn-positive cells in the SNpc are shown in the right column of panel **D**. Scale bars: 50 μm. *n* = 4–6. **F**, **G** Immunohistochemical staining and quantification of TH-positive cells in the SNpc of WT and Cntnap4^+/−^ mice injected with either AAV-GFP or AAV-*h*α-Syn. Scale bars: 100 μm. Magnified images of TH-positive cells in the SNpc are shown in the right column of panel G. Scale bars: 50 μm; *n* = 6–10. **H**–**J** The protein expression levels of Cntnap4, *h*α-Syn, phosphorylation of α-synuclein at serine 129, and mouse α-synuclein were determined by western blotting; *n* = 3 per group. **K**, **L** Total distance traveled and movement speed in the open-field of WT and Cntnap4^+/−^ mice injected with either AAV-GFP or AAV-*h*α-Syn. **M** The rotarod test was used to examine the motor coordination of mice; *n* = 11, 11, 10, and 7 in the WT + AAV-GFP, WT + AAV-*h*α-Syn, Cntnap4^+/−^ + AAV-GFP, and Cntnap4^+/−^ + AAV-*h*α-Syn groups, respectively. Results are expressed as the mean ± SEM. **p < 0.01, *p < 0.05 vs^.^ WT; ^##^*p* < 0.01, ^#^*p* < 0.05 vs. AAV-*h*α-Syn; ^&&^*p* < 0^.^01, ^&^*p* < 0.05 vs. Cntnap4^+/−^. Statistical significance was determined using two-way ANOVA + Bonferroni’s multiple comparisons test.

To evaluate the impact of *Cntnap4* loss on α-synuclein pathology, we used heterozygous *Cntnap4* null mice established by our group. WT and Cntnap4^+/−^ mice received bilateral stereotaxic injections of either AAV-*h*α-Syn or AAV-GFP in the substantia nigra pars compacta (SNpc) for 8 weeks (Fig. 1A). We found that human α-synuclein was immunoreactive in the TH-positive neurons of mice injected with AAV-*h*α-Syn (Fig. 1B), and the number of α-synuclein immunoreactive cells was increased in the striatum and SNpc of Cntnap4^+/−^ + AAV-*h*α-Syn mice (Fig. 1C–E). AAV-*h*α-Syn induced nigrostriatal DA neuronal death and increased the protein expression level of human α-synuclein, as well as the phosphorylation of serine 129 of α-synuclein, which was the dominant pathological modification of α-synuclein [25], and was considerably more apparent in the SNpc of Cntnap4^+/−^ + AAV-*h*α-Syn mice (Fig. 1F–J, Fig. S1A–D). AAV-*h*α-Syn decreased synapsin III in the SN of Cntnap4^+/−^ mice, and both AAV-*h*α-Syn and Cntnap4^+/−^ reduced nigral PSD-95 expression, suggesting that these factors may affect synaptic plasticity (Fig. S2A–C).

*Cntnap4* partial deficiency decreased the total traveled distance, movement speed, and number of entries to the center zone in mice injected with AAV-*h*α-Syn in the open field test, and worsened the performance of AAV-*h*α-Syn mice in the rotarod test (Fig. 1K–M, Fig. S3A). In addition, both AAV-*h*α-Syn and Cntnap4^+/−^ reduced the performance of mice in the grasping test and led to spontaneous alterations in the Y maze, while other behavioral parameters showed no obvious changes (Fig. S3B–I). Thus, we successfully established an exogenous α-synuclein mouse model, in which *Cntnap4* partial deficiency aggravates α-synuclein pathology, nigral DA neuronal death, and impairs motor function.

### *Cntnap4* partial deficiency damages mitochondrial function and induces α-synuclein release via ferroptosis

**Fig. 2:**
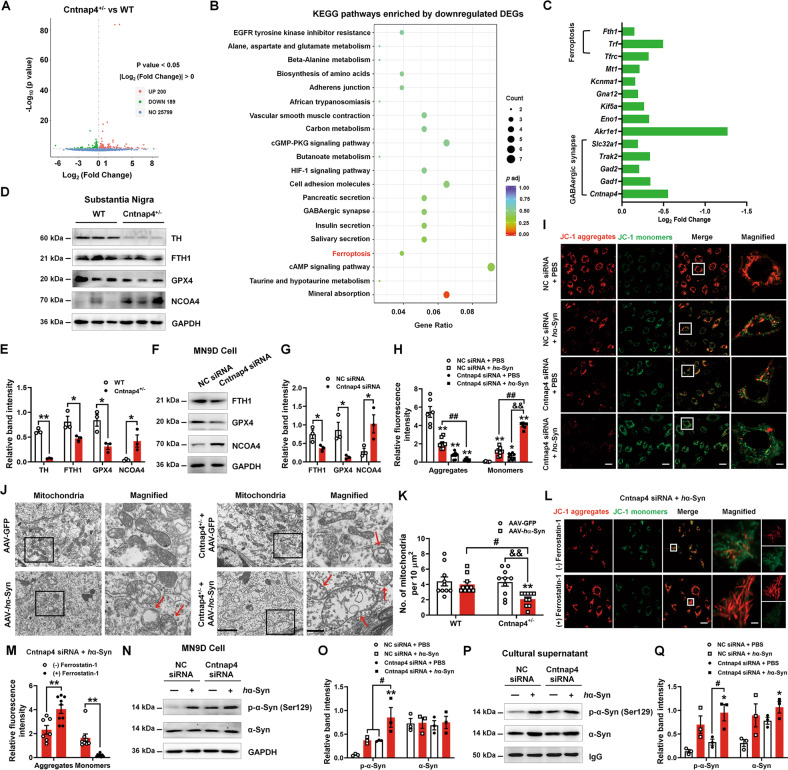
*Cntnap4* partial deficiency damages mitochondrial function and induces α-synuclein release via ferroptosis. **A** DEGs between Cntnap4^+/−^ and WT mice are shown in a volcano plot. **B** KEGG pathways enriched by downregulated DEGs between Cntnap4^+/−^ and WT mice. Note that ferroptosis is highlight in red. **C** Representative downregulated DEGs enriched in ferroptosis and GABAergic synapse. **D**, **E** The protein expression levels of TH, FTH1, GPX4, and NCOA4 in the SN were determined by western blotting; *n* = 3 per group. **F**, **G** The protein expression levels of FTH1, GPX4, and NCOA4 in MN9D cells treated with NC siRNA and Cntnap4 siRNA were determined by western blotting; *n* = 3 per group. **H**, **I** Immunofluorescence staining and quantification of JC-1 aggregates and monomers in MN9D cells treated with NC siRNA + PBS, NC siRNA + *h*α-Syn, Cntnap4 siRNA + PBS, and Cntnap4 siRNA + *h*α-Syn. Scale bars: 20 μm. Magnified figures are shown in the right column of panel **I**. Scale bars: 4 μm; *n* = 6–9. **J**, **K** Ultrastructural analysis of mitochondria in the SNpc of WT and Cntnap4^+/−^ mice injected with either AAV-GFP or AAV-*h*α-Syn. Scale bars, 2 μm, and 500 nm for magnified images. Red arrows show mitochondrial swelling and mitochondrial cristae disappearance. *n* = 10 per group. **L**, **M** Immunofluorescence staining and quantification of JC-1 aggregates and monomers in MN9D cells treated with Cntnap4 siRNA + *h*α-Syn plus ferrostatin-1. Scale bars: 20 μm. Magnified figures are shown in the right column of panel **L**. Scale bars: 2 μm; *n* = 7–9. **N**, **O** The protein expression levels of phosphorylated and mouse α-synuclein in MN9D cells treated with NC siRNA + PBS, NC siRNA + *h*α-Syn, Cntnap4 siRNA + PBS, and Cntnap4 siRNA + *h*α-Syn; *n* = 3 per group. **P**, **Q** Supernatant levels of phosphorylated and mouse α-synuclein from MN9D cells treated with NC siRNA + PBS, NC siRNA + *h*α-Syn, Cntnap4 siRNA + PBS, and Cntnap4 siRNA + *h*α-Syn; *n* = 3 per group. Results are expressed as the mean ± SEM. **p < 0.01, *p < 0.05 vs^.^ WT or NC siRNA; ^##^*p* < 0.01, ^#^*p* < 0.05 vs. AAV-*h*α-Syn or NC siRNA + *h*α-Syn; ^&&^*p* < 0.01 vs. Cntnap4^+/−^ + AAV-*h*α-Syn or Cntna*p*4 siRNA + PBS. Statistical significance was determined using Student’s *t*-test (**E**, **G**, **M**), one-way ANOVA + Tukey’s multiple comparisons test (**H**, **O**, **Q**), and two-way ANOVA + Bonferroni’s multiple comparisons test (**K**).

We first sought to describe the altered signaling pathways between Cntnap4^+/−^ and WT mice. Consistent with previous study [26], we found downregulated differentially expressed genes (DEGs) enriched in the GABAergic synapse (Fig. 2A–C). Notably, we found that several downregulated DEGs (*Fth1*, *Trf*, and *Tfrc*) were enriched in ferroptosis (Fig. 2B, C). We confirmed this result by showing decreased expression of ferritin heavy chain 1 (FTH1), glutathione peroxidase 4 (GPX4), and increased nuclear receptor coactivator 4 (NCOA4) in the SN of Cntnap4^+/−^ mice and dopaminergic MN9D cells treated by Cntnap4 siRNA (Fig. 2D–G). Because dysfunctional mitochondria play critical roles in signaling for ferroptosis [27], double treatment of Cntnap4 siRNA and human α-synuclein fibrils (*h*α-Syn) hindered aggregated JC-1 within the mitochondrial matrix to form JC-1 aggregates, and thus increased JC-1 monomers, compared to single Cntnap4 siRNA or *h*α-Syn treatment (Fig. 2H, I). Ultrastructural results revealed an apparent reduction in the number of mitochondria and mitochondrial morphological changes (mitochondrial swelling and disappearance of cristae) in the nigral DA terminal of Cntnap4^+/−^ + AAV-*h*α-Syn mice compared to AAV-*h*α-Syn or Cntnap4^+/−^ mice (Fig. 2J, K). Remarkably, the ferroptosis inhibitor, ferrostatin-1, efficiently rescued the imbalance between JC-1 aggregates and monomers in MN9D cells treated with Cntnap4 siRNA and *h*α-Syn, suggesting that ferrostatin-1 improves the damaged mitochondrial membrane potential (Fig. 2L, M). We further confirmed that *Cntnap4* knockdown in *h*α-Syn-treated cells exacerbated intracellular α-synuclein pathology and induced pathological α-synuclein release in the culture supernatant (Fig. 2N–Q). These observations suggest that ferroptosis induced by dysfunctional mitochondrial function may underly insufficient *Cntnap4* exacerbated parkinsonian phenotypes.

### *Cntnap4* partial deficiency elicits inflammatory response in mice with α-synuclein burden

**Fig. 3:**
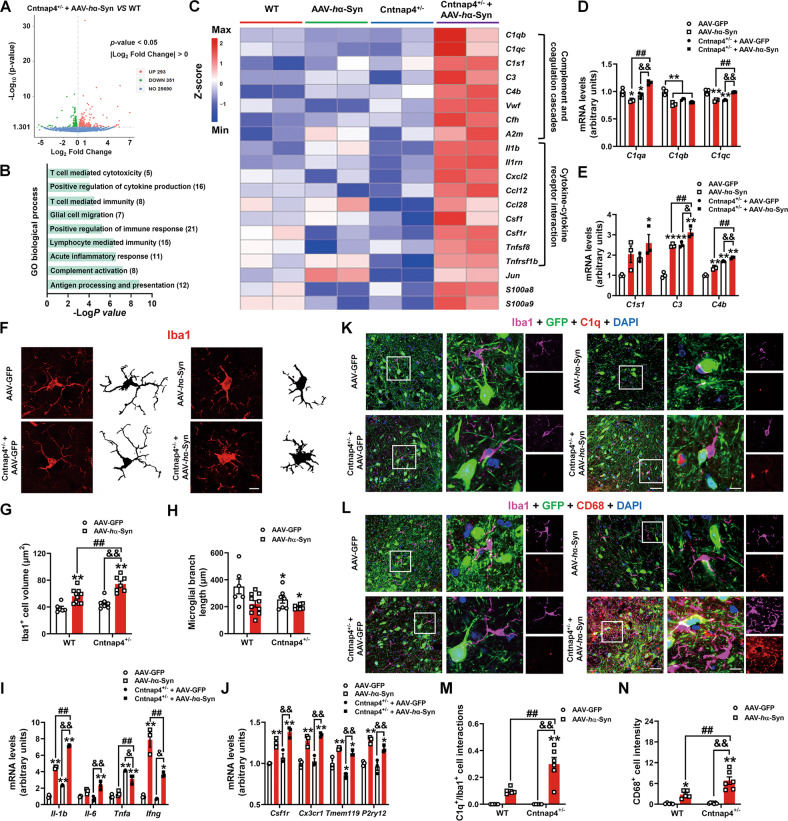
*Cntnap4* partial deficiency induces pro-inflammatory response in mice with α-synuclein burden. **A** DEGs between Cntnap4^+/−^ + AAV-*h*α-Syn and WT mice were shown in a volcano plot. **B** GO pathways enriched by upregulated DEGs between Cntnap4^+/−^ + AAV-*h*α-Syn and WT mice. **C** Representative DEGs among WT, AAV-*h*α-Syn, Cntnap4^+/−^, and Cntnap4^+/−^ + AAV-*h*α-Syn mice are shown in a heatmap. Note that the genes *C1qb*, *C1qc*, *C1s1*, *C3*, *C4b*, *Vwf*, *Cfh*, and *A2m* were enriched in the “complement and coagulation cascades” pathway, *Il1b*, *Il1rn*, *Cxcl2*, *Ccl12*, *Ccl28*, *Csf1*, *Csf1r*, *Tnfsf8*, and *Tnfrsf1b* were enriched in “Cytokine-cytokine receptor interaction” pathway. **D**, **E** The mRNA expression levels of *C1qa*, *C1qb*, *C1qc*, *C1s1*, *C3*, and *C4b* were determined by qRT-PCR. *n* = 3 per group. **F** Immunofluorescence staining of Iba1-positive cells in the SNpc of WT and Cntnap4^+/−^ mice injected with either AAV-GFP or AAV-*h*α-Syn. Scale bars: 30 μm. Skeletal images are shown in the right column. **G**, **H** Quantification of the volume and branch length of Iba1-positive cells in (**F**); *n* = 6–10. **I**, **J** The mRNA expression levels of *Il-1b*, *Il-6*, *Tnfa*, *Ifng*, *Csf1r*, *Cx3cr1*, *Tmem119*, and *P2ry12* were determined by qRT-PCR; *n* = 3 per group. **K**, **L** Co-staining of Iba1 with C1q (**K**) and CD68 (**L**) in the SNpc of WT and Cntnap4^+/−^ mice injected with either AAV-GFP or AAV-*h*α-Syn. Scale bars: 40 μm. Magnified images are shown in the right column. Scale bars: 8 μm. **M**, **N** Quantification of the interaction area of Iba1 with C1q (**K**) and CD68 positive cell intensity (**L**); *n* = 5–7. Results are expressed as the mean ± SEM. **p < 0.01, *p < 0.05 vs^.^ WT; ^##^*p* < 0.01 vs. AAV-*h*α-Syn; ^&&^*p* < 0.01, ^&^*p* < 0.05 vs. Cntnap4^+/−^. Statistical significance was determined using two-way ANOVA + Bonferroni’s multiple comparisons test.

Subsequently, we compared the DEGs between Cntnap4^+/−^ + AAV-*h*α-Syn and WT mice (Fig. 3A). The upregulated DEGs were enriched in inflammation-associated signaling pathways, such as “antigen processing and presentation,” “complement activation,” “acute inflammatory response,” and “positive regulation of cytokine production” (Fig. 3B). We then listed the representative DEGs enriched in the top hit of inflammatory pathways in the SN of Cntnap4^+/−^ + AAV-*h*α-Syn compared to the other three groups, which included “complement and coagulation cascades” (*C1qb*, *C1qc*, *C1s1*, *C3*, *C4b*, *Vwf*, *Cfh*, and *A2m*) and “cytokine-cytokine receptor interaction” (*Il1b*, *Il1rn*, *Cxcl2*, *Ccl12*, *Ccl28*, *Csf1*, *Csf1r*, *Tnfsf8*, and *Tnfrsf1b*) (Fig. 3C). Notably, the upregulated DEGs between Cntnap4^+/−^ + AAV-*h*α-Syn and Cntnap4^+/−^/AAV-*h*α-Syn groups were commonly enriched in “complement and coagulation cascades” (Figs. S4 and S5). We further verified the complement-related gene expression by qRT-PCR and found that the expression of *C1qa*, *C1qc*, *C3*, and *C4b* were actually increased in Cntnap4^+/−^ + AAV-*h*α-Syn mice compared to others (Fig. 3D, E). We next tested whether an inflammatory reaction was activated in Cntnap4^+/−^ + AAV-*h*α-Syn mice. The immunofluorescence staining demonstrated an increased microglial volume and a reduction in microglial process complexity in the SNpc of Cntnap4^+/−^ + AAV-*h*α-Syn mice compared to the other groups (Fig. 3F–H), indicating that the microglia change from a “resting” ramified phenotype to an “activated” bushy phenotype. Compared to AAV-*h*α-Syn, Cntnap4^+/−^ + AAV-*h*α-Syn increased *Il-1b* and *Tnfa* mRNA expression levels, as well as serum IL-6 and granulocyte colony-stimulating factor (G-CSF) protein expression levels (Fig. 3I and Figs. S6A and B). Cntnap4^+/−^ + AAV-*h*α-Syn increased the *Il-1b*, *Il-6*, *Ifng*, *Csf1r*, *Cx3cr1*, *Tmem119*, and *P2ry12* mRNA expression levels compared to the Cntnap4^+/−^ mice (Fig. 3I, J). However, AAV-*h*α-Syn or Cntnap4^+/−^ showed no obvious effects on other serum cytokines, included IL-2, IL-10, and IL-17 (Fig. S6C–L and Fig. S7). We validated significant activation of the inflammatory response in the SNpc of Cntnap4^+/−^ + AAV-*h*α-Syn by co-staining Iba1 with C1q and CD68 (microglial activation makers) (Fig. 3K–N). We also found that Iba1-positive cells moved close to tyrosine hydroxylase (TH)-positive cells, while C1q-positive cells colocalized with *h*α-Syn burden (Fig. S8A–D).

The downregulated DEGs between Cntnap4^+/−^ + AAV-*h*α-Syn and Cntnap4^+/−^/AAV-*h*α-Syn groups were enriched in “dopaminergic synapse” and “synaptic vesicle cycle” pathways (Fig. S9A–D). Our ultrastructural results also indicate the decreased nigral synaptic vesicles in the Cntnap4^+/−^, AAV-*h*α-Syn, and Cntnap4^+/−^ + AAV-*h*α-Syn mice (Fig. S10). These data reveal the severe nigral inflammatory response in the Cntnap4^+/−^ + AAV-*h*α-Syn mice.

### Overexpression of α-synuclein in *Cntnap4* partial deficient mice sharpens inflammation by activating the astrocyte–microglia C3-C3aR pathway

**Fig. 4:**
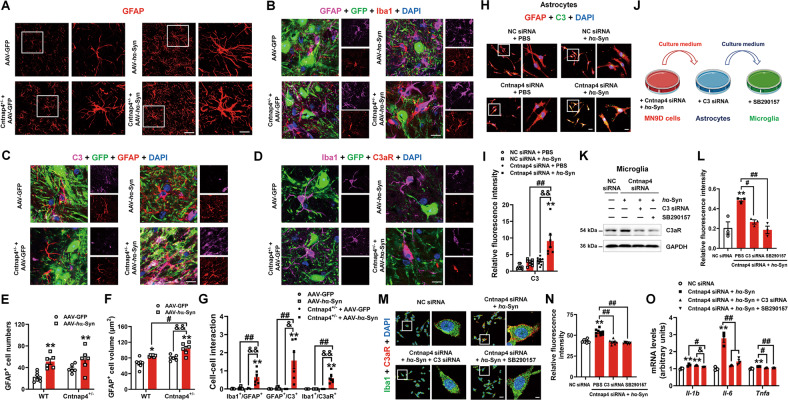
Activation of the astrocyte–microglia C3-C3aR pathway in *Cntnap4* partial deficient *mice* with α-synuclein overexpression. **A** Immunofluorescence staining of GFAP-positive cells in the SNpc of WT and Cntnap4^+/−^ mice injected with either AAV-GFP or AAV-*h*α-Syn. Scale bars: 20 μm. Magnified images of GFAP-positive cells in the SNpc are shown in the right column of panel A. Scale bars: 5 μm. **B**–**D** Co-staining of GFAP with Iba1 (**B**), C3 with GFAP (**C**), and Iba1 with C3aR (**D**) in the SNpc of WT and Cntnap4^+/−^ mice injected with either AAV-GFP or AAV-*h*α-Syn. Scale bars: 40 μm. Magnified images are shown in the right column. Scale bars: 8 μm. **E**, **F** Quantification of the cell number and volume of GFAP-positive cells in (**A**); *n* = 6–7. **G** Quantification of the interaction area of GFAP with Iba1 (**B**), C3 with GFAP (**C**), and Iba1 with C3aR (**D**); *n* = 5–7. **H** Co-staining of GFAP with C3 in astrocytes treated with the culture supernatant from MN9D cells (NC siRNA + PBS, NC siRNA + *h*α-Syn, Cntnap4 siRNA + PBS and Cntnap4 siRNA + *h*α-Syn). **I** Quantification of C3 intensity in panel (**H**); *n* = 7–9. **J** Schematic model showing the culture medium from Cntnap4^+/−^ or/both AAV-*h*α-Syn-treated MN9D cells to treat primary astrocytes. Then, the culture supernatant from astrocytes was used to treat primary microglia. C3 siRNA was used to treat astrocytes and SB290157 was used to treat microglia. **K**, **L** Microglia were treated with the culture supernatant from Cntnap4 siRNA + *h*α-Syn-treated astrocytes with C3 siRNA and SB290157. The protein expression level of C3 was determined by western blotting; *n* = 3 per group. **M**, **N** Co-staining of Iba1 with C3aR in the microglia from panel K; *n* = 8–11. **O** The mRNA expression levels of *Il-1b*, *Il-6*, and *Tnfa* in the microglia from panel (**K**) were determined by qRT-PCR; *n* = 3 per group. Results are expressed as the mean ± SEM. **p < 0.01, *p < 0.05 vs^.^ WT or NC siRNA; ^##^*p* < 0.01, ^#^*p* < 0.05 vs. AAV-*h*α-Syn, *h*α-Syn or Cntnap4 siRNA + *h*α-Syn; ^&&^*p* < 0.01, ^&^*p* < 0.05 vs. Cntnap4^+/−^, Cntnap4 siRNA, or Cntnap4 siRNA + *h*α-Syn + C3 siRNA. Statistical significance was determined using two-way ANOVA + Bonferroni’s multiple comparisons test (**E**–**G**), and one-way ANOVA + Tukey’s multiple comparisons test (**I**, **L**, **N**, **O**).

Our RNA-seq data suggest the increase in complement-related genes (*C1q*, *C3*, and *C4*) in Cntnap4^+/−^ + AAV-*h*α-Syn mice. Previously, a striking physical interaction between astrocytes and microglia mediated by C3-C3aR signaling was reported in murine models of neuromyelitis optica and epilepsy [28, 29]. Therefore, we next sought to determine whether astrocytes also active microglia in this manner. Intriguingly, the number of GFAP-positive cells was significantly increased, their soma volume was greater, and their processes were thicker in the Cntnap4^+/−^ + AAV-*h*α-Syn group compared to those in the other groups (Fig. 4A, E, F), suggesting astrocyte activation. Surprisingly, in contrast to the other groups, astrocytes merged with microglia in the SNpc of Cntnap4^+/−^ + AAV-*h*α-Syn group (Fig. 4B, G). Being the main source of C3 in the brain, AAV-*h*α-Syn significantly induced activated astrocytes to produce extracellular C3 in Cntnap4^+/−^ mice (Fig. 4C, G). Astrocytic C3 can bind with the C3a receptor to induce an inflammatory reaction [28, 30]. We found that the microglia in the Cntnap4^+/−^ + AAV-*h*α-Syn mice exhibited striking C3aR upregulation (Fig. 4D, G). We then used the culture supernatant from Cntnap4^+/−^ and/or *h*α-Syn-treated MN9D cells to treat primary astrocytes. Consistently, our in vitro results suggest that the culture supernatant from *Cntnap4* knockdown in *h*α-Syn-treated cells significantly increased astroglial C3 expression levels (Fig. 4H, I, Fig. S11A). We further used the culture supernatant from astrocytes to treat primary microglia and found it to promote C3aR expression (Fig. 4J–N). Importantly, both C3 siRNA and C3aR antagonist (SB290157) abolished the increased C3aR and pro-inflammatory genes (*Il-1b*, *Il-6* and *Tnfa*) expression in microglia treated with the culture supernatant from astrocytes (Fig. 4K–O, Fig. S11B). However, using *h*α-Syn or the culture supernatant from Cntnap4^+/−^ or/both AAV-*h*α-Syn-treated MN9D cells did not induce C3aR expression in microglia, suggesting C3 is actually derived from the astrocytes (Fig. S12A–F).

These observations demonstrate that α-synuclein overexpression in Cntnap4^+/−^ mice promotes astrocyte–microglia interplay through the C3-C3aR pathway to induce an inflammatory response.

### Eliminating microglia ameliorates AAV-*h*α-Syn-induced parkinsonian lesions in the *Cntnap4* partial deficient mice by interrupting astrocyte–microglia interplay

**Fig. 5:**
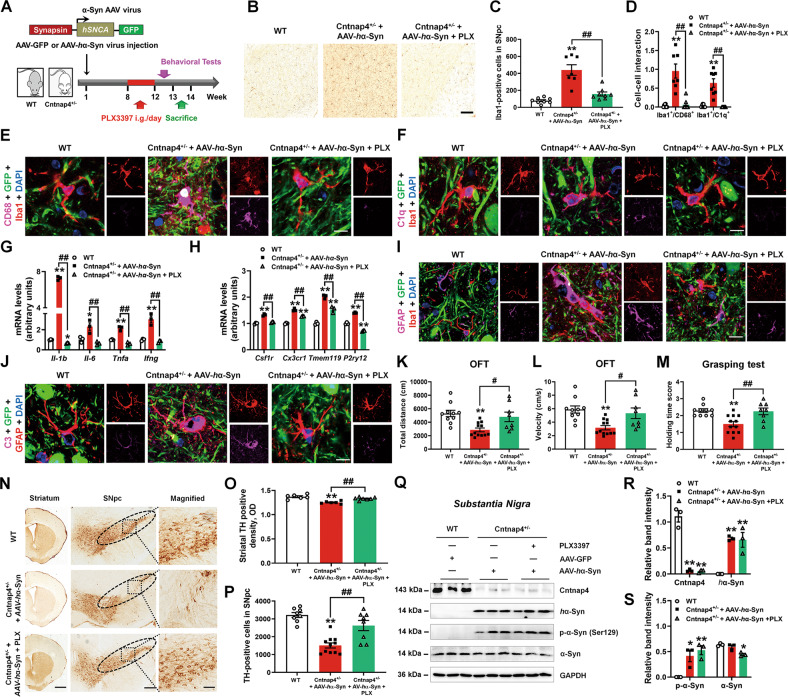
Eliminating microglia ameliorates AAV-*h*α-Syn-induced parkinsonian lesions in the *Cntnap4* partial deficient mice. **A** Schematic model of the study design. **B**, **C** Immunohistochemical staining and quantification of Iba1-positive cells in the SNpc of WT, Cntnap4^+/−^ + AAV-*h*α-Syn, and Cntnap4^+/−^ + AAV-*h*α-Syn + PLX3397 groups. Scale bars: 50 μm. **D**–**F** Co-staining and quantification of Iba1 with CD68 (**E**), and Iba1 with C1q (**F**) in the SNpc of WT, Cntnap4^+/−^ + AAV-*h*α-Syn, and Cntnap4^+/−^ + AAV-*h*α-Syn + PLX3397 groups. Scale bars: 40 μm. Magnified images are shown in the right column of panel. Scale bars: 8 μm; *n* = 7–9. **G**, **H** The mRNA expression levels of *Il-1b*, *Il-6*, *Tnfa*, *Ifng*, *Csf1r*, *Cx3cr1*, *Tmem119*, and *P2ry12* were determined by qRT-PCR; *n* = 3 per group. **I**, **J** Co-staining of GFAP with Iba1 (**I**), and GFAP with C3 (**J**) in the SNpc of WT, Cntnap4^+/−^ + AAV-*h*α-Syn, and Cntnap4^+/−^ + AAV-*h*α-Syn + PLX3397 groups. Scale bars: 8 μm. **K**, **L** Total distance traveled and movement speed in WT, Cntnap4^+/−^ + AAV-*h*α-Syn, and Cntnap4^+/−^ + AAV-*h*α-Syn + PLX3397 groups. **M** The grasping test was used to examine the grip strength of mice; *n* = 10, 12, and 8 in the WT, Cntnap4^+/−^ + AAV-*h*α-Syn, and Cntnap4^+/−^ + AAV-*h*α-Syn + PLX3397 groups, respectively. **N**–**P** Immunohistochemical staining and quantification of TH-positive cells in WT, Cntnap4^+/−^ + AAV-*h*α-Syn, and Cntnap4^+/−^ + AAV-*h*α-Syn + PLX3397 groups. Scale bars: 1 mm for striatum, 100 μm for SNpc. Magnified images of TH-positive cells in the SNpc are shown in the right column. Scale bars: 50 μm; *n* = 6–11. **Q**–**S** Protein expression levels of Cntnap4, *h*α-Syn, phosphorylation of α-synuclein at serine 129, and mouse α-synuclein were determined using western blotting; *n* = 3 per group. Results are expressed as the mean ± SEM. **p < 0.01, *p < 0.05 vs^.^ WT; ^##^*p* < 0.01, ^#^*p* < 0.05 vs. Cntnap4^+/−^ + AAV-*h*α^-^Syn. Statistical significance was determined using one-way ANOVA + Tukey’s multiple comparisons test.

We next investigated whether interruption of astrocyte-microglia C3-C3aR pathway could rescue AAV-*h*α-Syn-induced parkinsonian damages in Cntnap4^+/−^ mice. To do this, we fed the Cntnap4^+/−^ + AAV-*h*α-Syn mice with PLX3397, an inhibitor of the CSF1 receptor [31], to eliminate microglia (Fig. 5A). Histopathological staining showed that PLX3397 significantly eliminated the nigral resident microglia in Cntnap4^+/−^ + AAV-*h*α-Syn mice (Fig. 5B, C). Elimination of microglia decreased the expression of two microglial activation markers, CD68 and C1q, in the SNpc of Cntnap4^+/−^ + AAV-*h*α-Syn mice (Fig. 5D–F). Deletion of microglia also significantly reduced the mRNA expression levels of pro-inflammatory cytokines (*Il-1b*, *Il-6*, *Tnfa*, and *Ifng*) and microglial homeostatic markers (*Csf1r*, *Cx3cr1*, *Tmem119*, and *P2ry12*) in the Cntnap4^+/−^ + AAV-*h*α-Syn mice (Fig. 5G, H). These data suggest that the elimination of microglia attenuates the immune response caused by AAV-*h*α-Syn in Cntnap4^+/−^ mice. Next, we examined the effects of microglial elimination on the C3-C3aR pathway. We found microglial depletion interrupted the interaction between astrocytes and microglia, and reduced the astrocytic production of C3 in the SNpc of Cntnap4^+/−^ + AAV-*h*α-Syn mice (Fig. 5I, J and Fig. S13A and B). Notably, elimination of microglia attenuated the movement disorder in the Cntnap4^+/−^ + AAV-*h*α-Syn mice in the OFT and grasping test (Fig. 5K–M), improved nigrostriatal DA neuronal death (Fig. 5N–P), and slightly decreased the endogenous α-synuclein level (Fig. 5Q–S). However, it had no apparent effects on the expression of exogenous and mouse phosphorylated α-synuclein in the Cntnap4^+/−^ + AAV-*h*α-Syn mice (Fig. 5Q–S). We also found that PLX3397 decreased the expressions of cytokines, such as *Il-1b*, *Il-6*, *Ifng*, *Csf1r*, *Cx3cr1*, *Tmem119*, and *P2ry12* as compared with AAV-*h*α-Syn mice, suggesting it may disturb the activation of microglia in the nigra of AAV-*h*α-Syn-injected mice (Fig. S14A and B). Besides, we also noticed that PLX3397 rescued nigral DA neuronal death in AAV-*h*α-Syn-injected mice (Fig. S14C).

Therefore, our results suggest that pharmacological suppression of microglia attenuates the immune reaction, motor impairment and DA neuronal death in Cntnap4^+/−^ + AAV-*h*α-Syn mice possibly through astrocyte–microglia interaction.

### *Cntnap4* knockdown in A53T α-synuclein mice reproduces pro-inflammatory response by activating the C3-C3aR pathway

**Fig. 6:**
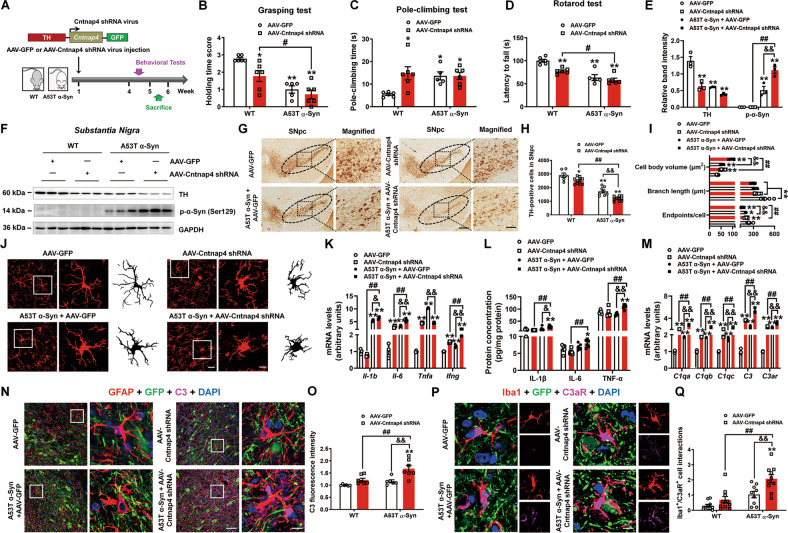
*Cntnap4* knockdown in A53T α-synuclein mice induces a pro-inflammatory response by activating the C3-C3aR pathway. **A** Schematic model of the study design. **B** The grasping test was used to examine the grip strength of mice. **C** The pole-climbing test was used to examine bradykinesia in the mice. **D** The rotarod test was used to examine the motor coordination of mice; *n* = 6, 6, 5, and 6 in the WT + AAV-GFP, WT + AAV-Cntnap4 shRNA, A53T α-Syn + AAV-GFP, and A53T α-Syn + AAV-Cntnap4 shRNA groups, respectively. **E**, **F** The protein expression levels of TH and phosphorylation of α-synuclein at serine 129 in the SN were determined by western blotting; *n* = 3 per group. **G**, **H** Immunohistochemical staining and quantification of TH-positive cells in the SNpc. Scale bars: 100 μm. Magnified images are shown in the right column. Scale bars: 50 μm; *n* = 6–10. **I**, **J** Immunofluorescence staining and quantification of Iba1-positive cells in the SNpc. Scale bars: 30 μm. Magnified images are shown in the middle column, and skeletal diagrams of Iba1-positive cells are shown in the right panel. Scale bars: 8.5 μm; *n* = 5–7. **K** The mRNA expression levels of *Il-1b*, *Il-6*, *Tnfa*, and *Ifng* were determined by qRT-PCR; *n* = 3 per group. **L** The protein expression of nigral Il-1β, IL-6, and TNF-α; *n* = 6 per group. **M** The mRNA expression levels of nigral *C1qa*, *C1qb*, *C1qc*, *C3*, and *C3ar* were determined by qRT-PCR; *n* = 3 per group. **N**, **O** Co-staining and quantification of GFAP with C3 in the SNpc. Scale bars: 40 μm. Magnified images are shown in the right column of panel N. Scale bars: 8 μm; *n* = 6–7. **P**, **Q** Co-staining and quantification of Iba1 with C3aR in the SNpc. Scale bars: 8 μm; *n* = 9–10. Results are expressed as the mean ± SEM. ^**^p < 0.01, ^*^p < 0.05 vs^.^ WT; ^##^*p* < 0.01, ^#^*p* < 0.05 vs. AAV-Cntnap4 shRNA; ^&&^*p* < 0.01, ^&^*p* < 0.05 vs. A53T α-Syn. Statistical significance was determined using two-way ANOVA + Bonferroni’s multiple comparisons test.

Given that abnormal astrocyte-microglia communication ignites the inflammatory response in Cntnap4^+/−^ + AAV-*h*α-Syn mice, we next evaluated whether this pathway is activated in A53T α-synuclein (A53T α-Syn) mice injected with AAV-Cntnap4 shRNA (Fig. 6A). A53T α-Syn + AAV-Cntnap4 shRNA exacerbated the motor dysfunction compared to AAV-Cntnap4 shRNA, but showed no obvious difference with A53T α-Syn (Fig. 6B–D). However, we observed considerably more obvious phosphorylated α-synuclein in the SN of A53T α-Syn + AAV-Cntnap4 shRNA mice compared to AAV-Cntnap4 shRNA/A53T α-Syn, though TH-positive cells appeared to decrease further in A53T α-Syn + AAV-Cntnap4 shRNA mice (Fig. 6E–H). In response to the strong α-synuclein pathology, the microglia were further activated, manifesting the morphological changes to an “activated” bushy phenotype, with increased mRNA and protein expression of pro-inflammatory cytokines (IL-1β, IL-6 and TNF-α) (Fig. 6I–L). Intriguingly, the levels of complement-related genes (*C1qa*, *C1qb*, *C1qc*, *C3*, and *C3ar*) were considerably more evident in A53T α-Syn + AAV-Cntnap4 shRNA mice compared to AAV-Cntnap4 shRNA/A53T α-Syn (Fig. 6M). Remarkably, we noticed enhanced astroglial C3 and microglial C3aR expression in the SNpc of A53T α-Syn + AAV-Cntnap4 shRNA mice compared to AAV-Cntnap4 shRNA/A53T α-Syn (Fig. 6N–Q). These findings suggest *Cntnap4* knockdown also activates pro-inflammatory response via the C3-C3aR pathway in A53T α-synuclein mice.

### Microglial delivery of C3aR antagonist improves the parkinsonian phenotype in A53T α-Syn mice injected with AAV-Cntnap4 shRNA

**Fig. 7:**
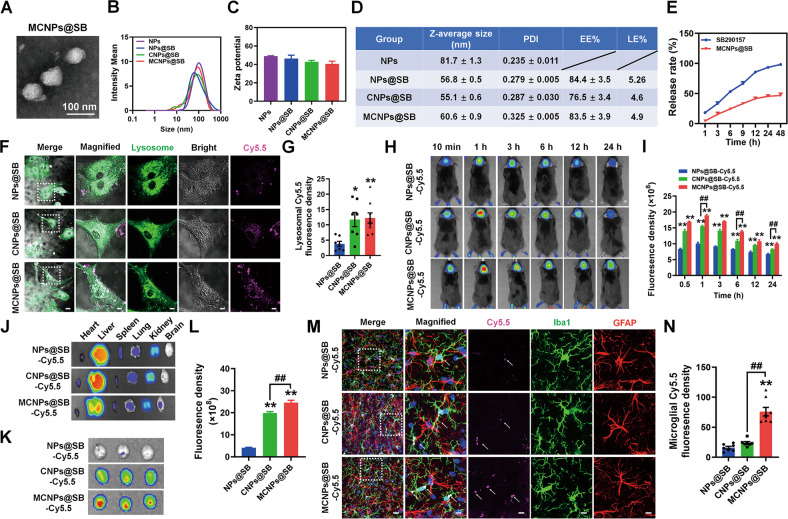
Establishment of the microglial C3aR antagonist delivery system. **A** Representative TEM images of MCNPs@SB. Scale bars: 100 nm. **B**, **C** Particle size and Zeta potential analysis of NPs, NPs@SB, CNPs@SB, and MCNPs@SB; *n* = 3 per group. **D** Z-average size, PDI, EE% and LE% of NPs, NPs@SB, CNPs@SB and MCNPs@SB; *n* = 3 per group. **E** The drug release kinetics of SB290157 and MCNPs@SB; *n* = 3 per group. **F**, **G** Representative images and quantitative fluorescence analysis of lysosomal tracker (Lyso-tracker) in microglia after 3 h incubation with Cy5.5-labeled NPs@SB, CNPs@SB, and MCNPs@SB; *n* = 7–9. Scale bars: 20 μm. Magnified images are shown in the right columns of panel. Scale bars: 6.5 μm. **H** Real-time fluorescence imaging of mice after intravenous injection of Cy5.5-labeled NPs@SB, CNPs@SB, and MCNPs@SB. **I** The fluorescence density in the brain at different time points; *n* = 3 per group. **J**, **K** Ex vivo imaging and corresponding fluorescence analysis of sacrificed tissues (heart, liver, spleen, lung, kidney, and brain) at 24 h after intravenous injection of Cy5.5-labeled NPs@SB, CNPs@SB, and MCNPs@SB. **L** The fluorescence density in the brain at 24 h after intravenous injection of Cy5.5-labeled NPs@SB, CNPs@SB and MCNPs@SB; *n* = 3 per group. **M** Representative images of Iba1 and GFAP staining at 6 h post-injection in SNpc derived from mice treated with Cy5.5-labeled NPs@SB, CNPs@SB and MCNPs@SB. Scale bars: 20 μm. Magnified images are shown in the right column of the panels. Scale bars: 6.5 μm. White arrows in the enlarged details show the presence of nanoparticles in microglia. **N** Analysis of the fluorescence density of microglial Cy5.5; *n* = 7–8. Results are expressed as the mean ± SEM. **p < 0.01, *p < 0.05 vs^.^ NPs@SB; ^##^*p* < 0.01 vs. CNPs@SB. Statistical significance was determined using one-way ANOVA + Tukey’s multiple comparisons test.

Given that *Cntnap4* knockdown accelerates the inflammatory response in A53T α-synuclein mice via astrocyte release of C3, activating microglial C3aR, we next tested whether inhibition of C3aR could exert neuroprotective effects. Although SB290157 is a well-known C3aR antagonist, high-dose and chronic SB290157 treatment has off-target effects [32, 33]. Based on this, we established a microglial targeted system to efficiently deliver SB290157. We used DSPE-PEG2000-NHS to link two peptides, the CRT peptide (sequence: CRTIGPSVC), which is a transferrin receptor 1-targeted peptide that assists with crossing the blood-brain barrier (BBB) [34], and the MG1 peptide (sequence: CHHSSSAR), which can target microglia [35]. The infrared spectrum results showed the successful synthesis of DSPE-PEG-CRT and DSPE-PEG-MG1 (Fig. S15A–D and S16A–D). The resulting SB290157 delivery system (NPs@SB) containing CRT peptide was named CNPs@SB, while that containing CRT and MG1 peptides was named MCNPs@SB. Transmission electron microscopy (TEM) images of MCNPs@SB demonstrated that they were typically spherical in shape (Fig. 7A). The size distribution, Zeta potential, average hydrodynamic diameter, polydispersity index (PDI), encapsulation efficiency (EE%), loading efficiency (LE%), and release behaviors of NPs@SB, CNPs@SB, and MCNPs@SB are indicated in Fig. 7B–E and Fig. S17A–E. CNPs@SB and MCNPs@SB appeared to have similar uptake capacity in primary microglia (Fig. 7F, G). However, the real-time in vivo and ex vivo results suggest that MCNPs@SB have better brain targeting capability (Fig. 7H–L). Our immunofluorescence results also indicate that MCNPs@SB successfully penetrate the BBB and target microglia but not astrocytes (Fig. 7M, N). Thus, MCNP@SB could deliver SB290157 across the BBB and target microglia.

**Fig. 8:**
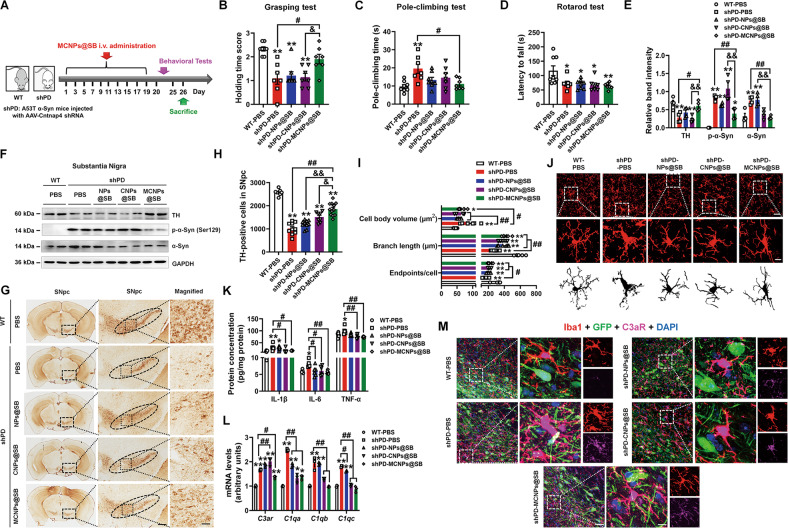
Microglial delivery of C3aR antagonist improves the parkinsonian phenotype in A53T α-Syn mice injected with AAV-Cntnap4 shRNA. **A** Schematic model of the study design. **B** The grasping test was used to examine the grip strength of mice. **C** The pole-climbing test was used to examine bradykinesia in the mice. **D** The rotarod test was used to examine the motor coordination of mice; *n* = 7–8. **E**, **F** The protein expression levels of TH, phosphorylation of α-synuclein at serine 129, and mouse α-synuclein in the SN were determined by western blotting; *n* = 3 per group. **G**, **H** Immunohistochemical staining and quantification of TH-positive cells in the SNpc. Scale bars: 100 μm. Magnified images are shown in the right column. Scale bars: 50 μm; *n* = 8–12. **I**, **J** Immunofluorescence staining and quantification of Iba1-positive cells in the SNpc. Scale bars: 30 μm. Magnified images are shown in the middle column, and skeletal diagrams of Iba1-positive cells are shown in the bottom panel. Scale bars: 8.5 μm; *n* = 6–7. **K** The protein expression of nigral Il-1β, IL-6, and TNF-α; *n* = 6 per group. **L** The mRNA expression levels of nigral *C3ar*, *C1qa*, *C1qb*, and *C1qc* were determined by qRT-PCR; *n* = 3 per group. **M** Co-staining and quantification of Iba1 with C3aR in the SNpc. Scale bars: 40 μm. Magnified images are shown in the right column of panel (**M**). Scale bars: 8 μm. Results are expressed as the mean ± SEM. **p < 0.01, *p < 0.05 vs^.^ WT; ^##^*p* < 0.01, ^#^*p* < 0.05 vs. shPD; ^&&^*p* < 0.01, ^&^*p* < 0.05 vs. shPD-MCNPs@SB. Statistical significance was determined using one-way ANOVA + Tukey’s multiple comparisons test.

We then used this strategy to deliver SB290157 to accurately block C3aR in microglia. NPs@SB, CNPs@SB, and MCNPs@SB (equivalent dose of SB290157) were used to treat A53T α-Syn mice injected with AAV-Cntnap4 shRNA (named shPD in this study) (Fig. 8A). Here, MCNPs@SB attenuated the motor dysfunction in the grasping and pole-climbing test, but not in the rotarod test (Fig. 8B–D). Notably, MCNPs@SB improved DA neuronal death and decreased the nigral phosphorylated and endogenous α-synuclein expression in shPD (Fig. 8E–H). MCNPs@SB also restored the bushy microglia towards ramified state (Fig. 8I, J), and suppressed the protein expression of IL-1β, IL-6, and TNF-α (Fig. 8K), suggesting MCNPs@SB exerts anti-inflammatory effects in shPD. MCNPs@SB inhibited the mRNA expression of nigral *C3ar*, *C1qa*, *C1qb*, and *C1qc*, and efficiently abolished the microglial C3aR expression in the SNpc of shPD (Fig. 8L, M, Fig. S18). Hence, microglial targeted delivery of C3aR antagonist suppresses the pro-inflammatory response in A53T α-Syn mice lacking *Cntnap4*.

## Discussion

Cntnap4 is highly enriched in cortical interneurons and nigral DA neurons. It is genetically linked with autism and functionally associated with GABAergic transmission [26, 36]. We previously revealed the crucial role of Cntnap4 in regulating DA neuron activity and fear memory processing [24, 37], and it has also been linked with PD and other aging-related diseases [24, 38]. Mechanistically, we found that *Cntnap4* partial deficiency induced nigral DA neuronal death, α-synuclein pathology, and motor dysfunction by damaging mitochondrial function [24]. In this study, we report that mice lacking *Cntnap4* induces a more severe parkinsonian phenotype in α-synuclein mouse models. Because Cntnap4 is not expressed in astrocytes or microglia [26, 36], we conclude that these defects may be derived from *Cntnap4* partial deficiency-induced diseased DA neurons that are susceptible to exogenous α-synuclein deposition. These duplicated effects mediate DA neuronal death and α-synuclein release. Then, the released α-synuclein induces astrocyte–microglia crosstalk and inflammatory reaction.

First, we investigated how *Cntnap4* partial deficiency damages DA neurons and induces α-synuclein release. Our results suggest that *Cntnap4* partial deficiency-induced DA neuronal death is associated with ferroptosis. Indeed, recent findings have revealed a critical role of ferroptosis in PD pathogenesis [39]. As an iron-dependent cell death pathway, ferroptosis involves nigral iron overload, glutathione depletion, lipid peroxidation, and elevated reactive oxygen species generation [40–42], all of which are well-known contributing factors to DA neuronal death. Functionally linked with these mechanisms, mitochondrial dysfunction plays a vital role in ferroptosis [43, 44]. Previously, we found *Cntnap4* knockdown induces DA neuronal death by damaging mitochondrial function [24]. In this study, *Cntnap4* knockdown decreased the number of mitochondria and their membrane potential, which were rescued by using ferroptosis inhibitor. Our in vivo and in vitro results suggest that insufficient *Cntnap4* aggravated α-synuclein pathology, while α-synuclein release was also observed in *Cntnap4* siRNA and *h*α-Syn-treated cells. Thus, we conclude that *Cntnap4* partial deficiency in α-synuclein models promotes DA neuronal death possibly via ferroptosis induced by mitochondrial dysfunction. In this work, single injection of AAV-*h*α-Syn induced lower DA neuronal death compared to a previous study [45], possibly because our AAV-*h*α-Syn system did not contain the cytomegalovirus immediate-early (CMVie)-enhancer, which increases the expression of transduced proteins and related neurodegeneration. Therefore, we did not observe the astrocyte–microglia crosstalk in AAV-*h*α-Syn injected mice.

Second, we determined the interplay between astrocytes and microglia. In the brain, astrocytes communicate with microglia though signaling factors, such as neurotransmitters, cytokines, chemokines, and extracellular vesicles [46–48], which contribute to synapse development, pruning, and maintenance of the local environment [49, 50]. Over the last few decades, astrocyte–microglia crosstalk has been widely studied in neurological diseases [51]. Microglia augment astrocyte-mediated inflammatory activation in manganese exposure, traumatic brain injury, and stoke [46, 47, 52]. Microglia activation has also been found to convert astrocytes into a neurotoxic A1 phenotype in PD [53]. However, the mechanism underlying the contribution of their interplay to α-synuclein pathology remains poorly understood. We report that *Cntnap4* partial deficiency exacerbates α-synuclein pathology, which induces communication between astrocytes and microglia through the C3-C3aR signaling pathway. C3 complement is a crucial component of the innate immune system and, together with other complement proteins, forms a major host mechanism for the detection and clearance of potential pathogens. C3 is closely associated with PD pathogenesis, and high C3 levels in the serum and cerebrospinal fluid correlate with worse quality of life and memory ability in patients with PD [54–56]. Astrocytes are the main source of C3 in the central nervous system, and astroglial Kir6.1/K-ATP channel deficiency was previously reported to contribute to PD pathogenesis by inducing astrocyte–neuron interaction through C3-C3aR signaling [57]. Using α-synuclein preformed fibril-injected mice and human A53T α-Syn mice, Ma et al. identified that the complement and coagulation cascade pathways are the significant differential pathways compared to the controls [58]. Moreover, they found that C3-positive astrocytes were increased in the ventral midbrain of PD mice and that astrocyte-secreted C3 could induce DA neuron degeneration [58]. Intriguingly, similar to these observations, our results reveal that in the combination of *Cntnap4* partial deficiency and α-synuclein pathology models, the complement pathway is activated, alongside damaged nigral DA neuronal death and motor dysfunction. In these in vitro and in vivo models, astrocytes release C3, which activates microglial C3aR, leading to initiation of the downstream inflammatory pathway. Genetic knockdown and suppression of C3aR abolishes the inflammatory response and DA neuronal death. Thus, our study provides evidence that astrocyte–microglia C3-C3aR signaling is required for the *Cntnap4* partial deficiency*-*aggravated α-synuclein pathology.

Actually, we detected the increased mRNA expressions of *Il-1b*, *Ifng*, *Csf1r*, *Cx3cr1*, *Tmem119*, and *P2ry12* in AAV-*h*α-Syn-injected mice, and increased *Il-1b* and *Tnfa* expressions in Cntnap4^+/−^ mice (Fig. 3I, J). In addition, we also found AAV-*h*α-Syn enhanced the microglial volume and promoted the activation of microglia (Fig. 3F, L). These data suggest AAV-*h*α-Syn actually induces inflammation, consistent with previous study [22]. In this study, we mainly found *Cntnap4* partial deficiency aggravates α-synuclein pathology, which means this combined model (Cntnap4^+/−^ + AAV-*h*α-Syn) has much more severe α-synuclein pathology than AAV-*h*α-Syn or Cntnap4^+/−^ mice. By virtue of RNA-seq, we reveal the complement pathway is obvious activated and microglia-astrocyte crosstalk is emerging in this combined model. Thus, we conclude this crosstalk may be responsible for the severe α-synuclein pathology. However, we did not detect this phenomenon in the AAV-*h*α-Syn or Cntnap4^+/−^ mice, which may suggest the α-synuclein pathology and dopamine neuron death in these models may be not due to the crosstalk mediated by complement pathway. Regarding AAV-*h*α-Syn or Cntnap4^+/−^ mice showed no obvious effects on other serum cytokines, included IL-2, IL-10, and IL-17, we would like to say, on one hand, in some studies, these cytokines were reported unaltered in the serum of PD patients [59]; on the other hand, serum cytokines may not reflect the inflammatory state in the brain. Since we mainly focus on the complement pathway in this study, we may need further study to clarify the role of these cytokines.

To this end, we tested how the astrocyte–microglia communication could be manipulated in the context of PD? Recently, targeting the bidirectional signals between microglia and other neuronal cells has been considered an attractive therapeutic option for PD and other neurodegenerative diseases, for which several strategies have been developed [60–64]: (1) strategies to directly inhibit inflammation or pro-inflammatory cytokines, such as minocycline and NOD-like receptor protein 3 (NLRP3) inhibitor, effectively suppress microglial inflammation, nigral DA neuronal death, and α-synuclein pathology in PD [63, 65, 66]; (2) active or passive immunization to produce antibodies against α-synuclein and passive immunotherapy targeting α-synuclein have shown beneficial results in preclinical studies through the augmentation of α-synuclein clearance by microglia, with several immunotherapeutic strategies for PD currently in phase 2 clinical trials [62, 67, 68]; and (3) targeting bidirectional signals between astrocytes and microglia, such as the glucagon-like peptide 1 receptor agonist NLY01, has been revealed to protect DA neurons against inflammatory responses elicited by astrocytes and microglia [53]. Recently we developed a biomimetic strategy to implement accurate microglial delivery [69]. In this study, we adopted two strategies, including eliminating microglia using a CSF1R antagonist, PLX3397. Previously, using different α-synucleinopathy models of PD, some groups also report that PLX3397 could inhibit α-synuclein propagation via deleting microglia [70, 71]. In this study, we mainly focus on the astrocyte-microglia crosstalk mediated by overexpression of α-synuclein in *Cntnap4* partial deficient mice, and we want to explore whether deletion of microglia disrupts this interplay. Here we reported that microglial depletion reduces astrocyte–microglia interaction, inflammatory response, DA neuron loss, and motor dysfunction. However, more accurate therapies are in urgent need. Owing to the properties of immune cells, microglia are resistant to manipulation by recombinant viruses such as lentiviruses and adeno-associated viruses [72], while the BBB deters pharmacological treatment. To overcome these barriers, we developed a microglial targeted system to deliver the C3aR antagonist for intervention by successfully penetrating the BBB and targeting microglia. Importantly, microglial delivery of C3aR antagonist alleviated DA neuronal death and α-synuclein pathology by blocking the pro-inflammatory response. This study is the first to test the effect of the C3aR antagonist SB290157 in PD, and further study needs to evaluate its safety for clinical use.

## Conclusions

**Fig. 9:**
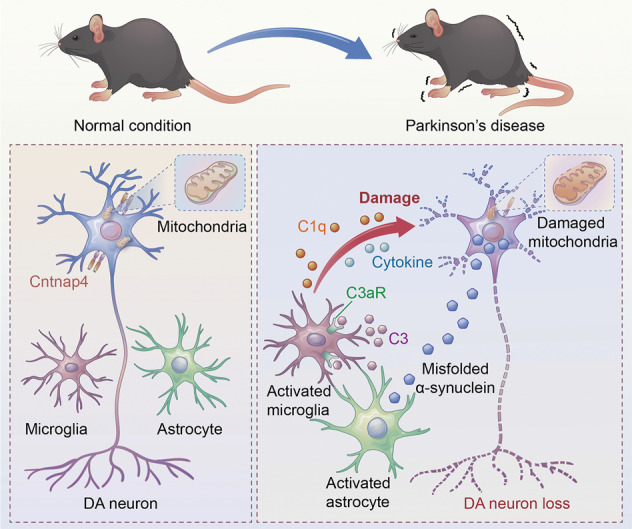
Schematic model of this study. *Cntnap4* partial deficiency augments DA neuronal death mediated by α-synuclein burden. Damaged DA neurons then release α-synuclein and induce the astrocyte–microglia crosstalk, which leads to astrocytic release of complement C3. Upon activation by the C3a receptor, microglia secretion of complement C1q and pro-inflammatory cytokines further drives DA neuronal death and aggravates motor dysfunction in PD.

Taken together, we provide evidence that *Cntnap4* partial deficiency accelerates α-synuclein pathology, nigrostriatal neuron degeneration, and motor disorders in α-synucleinopathy mouse models of PD. The astrocyte–microglia C3-C3aR signaling pathway was required for insufficient *Cntnap4*-exacerbated α-synuclein pathology, while microglia elimination and C3aR suppression attenuated these effects. Therefore, *Cntnap4* deficiency is critical to PD pathogenesis, and Cntnap4 merits further research as a therapeutic target for PD (Fig. 9).

## Materials and methods

### Cell culture

MN9D cells were purchased from American Type Culture Collection (ATCC, Manassas, VA, USA) and were cultured in Roswell Park Memorial Institute (RPMI) 1640 Medium (GIBCO, Carlsbad, CA, USA) supplemented with 10% fetal calf serum (FCS) and 1% penicillin/streptomycin at 37 °C in 5% CO_2_. Primary astrocytes and microglia were obtained according to our previous studies [73, 74]. Astrocytes were cultured in Dulbecco’s modified Eagle’s medium/F12 (DMEM/F12, GIBCO, Carlsbad, CA, USA) supplemented with 10% FCS, and microglia were cultured in DMEM/F12 supplemented with 10% FCS and GM-CSF at 37 °C in 5% CO_2_.

### Cntnap4 siRNA and C3 siRNA transfection

The siRNA sequence targeting *Cntnap4* (5′-GCTCAATAGTCAACTCTTT-3′) was chosen according to our previous study [24]. Three siRNAs targeting *C3* (siRNA-1, 5′- CCAAGAATCGCTACTTCCA-3′; siRNA-2, 5′-CCCTCATCATCTACCTAGA-3′; siRNA-3, 5′-CCGAGCTAACCAACATAGA-3′) were designed and synthesized by RioBio (Guangzhou, China). The siRNA transfection was performed as described previously [24].

### Cell treatment

MN9D cells were treated with Cntnap4 siRNA for 48 h, and 2 μg/μL human α-synuclein preformed fibrils (*h*α-Syn) were added for the last 24 h treatment. Then, the culture medium was collected and used to treat astrocytes for 3, 6, 12, and 24 h. Subsequently, *C3* mRNA expression was examined by quantitative reverse transcription polymerase chain reaction (qRT-PCR). To detect whether astroglial C3 activates microglial C3aR, the culture medium from astrocytes (12 h treatment) was collected to treat microglia for another 24 h. To examine the role of C3, astrocytes were treated with C3 siRNA (siRNA-1) for 48 h; and to examine the role of C3aR, microglial were treated with 1 μg/μL C3aR antagonist (SB290157) (S8931, Selleck, Houston, TX, USA) for 9 h. Subsequently, the microglia were collected and examined using western blotting, immunofluorescence analyses and qRT-PCR.

### Evaluation of α-synuclein released to the culture supernatant

To examine the α-synuclein released from MN9D cells treated with Cntnap4 siRNA and *h*α-Syn, 2 mL culture medium from the MN9D cells was collected and centrifuged at 7500 × *g* for 1 h at 4 °C by Microsep Advance Centrifugal Devices with Omega Membrane 3 K (#MCP003C41, Pall Corporation, NY, USA). The concentrated medium was then mixed with SDS-PAGE Sample Loading Buffer (Beyotime, Shanghai, China). Samples were boiled at 100 °C for 10 min and subjected to western blotting, with IgG set as the internal control.

### Animals

Adult (8-week-old) male C57BL/6J mice were purchased from SPF Biotechnology Co., Ltd. (Beijing, China). Heterozygous male *Cntnap4* null (Cntnap4^+/−^) mice (12–14 weeks) were obtained by mating *Cntnap4* knockout mice with wild-type (WT) C57BL/6 J mice. *Cntnap4* knockout mice with C57BL/6 J genetic background have been reported in our previous studies [24] and were generated by Shanghai Model Organisms Center, Inc. (Shanghai, China). The hSNCA*A53T-Tg mice (also called A53T α-Syn mice) were obtained from the Shanghai Model Organisms Center, Inc (Shanghai, China). Calculations for sample sizes were performed using an online sample size calculator (https://clincalc.com/stats/samplesize.aspx). The allocation of mice in each group were randomized and blinded. Age-and sex-matched littermates were used as controls. Animals were housed in a 12-h dark–light cycle and had free access to water and food. All animal experimental procedures were performed according to the guidelines of Institutional Animal Care and Use Committee of Guangzhou Medical University, National Institute of Health guidelines on the care and use of animals (NIH Publications No. 8023, revised 1978) and the Helsinki Declaration of 1975 (as revised in 2008) concerning Human and Animal Rights.

### AAV-virus generation and stereotaxic injection

AAV-*h*α-Syn virus has been described in previous studies [75, 76]. Generally, AAV9 virus encoding overexpression of either human wild-type α-synuclein or green fluorescent protein (GFP) was driven by the Syn I promoter and enhanced using the woodchuck hepatitis virus posttranscriptional regulatory element (WPRE). WT or Cntnap4^+/−^ mice were stereotaxically injected into the bilateral SNpc with either AAV-GFP or AAV-*h*α-Syn. The AAV-Cntnap4 virus was reported in our previous study [24]. WT or A53T α-Syn were stereotaxically injected into the bilateral SNpc with either AAV-GFP or AAV-Cntnap4 shRNA. Briefly, mice were anesthetized and fixed on a stereotactic frame (RWD Life Sciences Corp., China). AAV-GFP, AAV-*h*α-Syn-GFP or AAV-Cntnap4 shRNA (packaged by Sunbio Medical Biotechnology, Shanghai, China) in 0.5 μL volume were injected into the bilateral SNpc at the target site, as reported previously (Bregma AP, −3.0 mm, ML, ±1.3 mm, DV, −4.7 mm) [75]. The syringe was left in place for 5 min before being slowly withdrawn.

### Animal administration

To detect the effects of AAV-*h*α-Syn on Cntnap4^+/−^ mice, mice were administered AAV-GFP or AAV-*h*α-Syn, and behavioral tests were performed 8 weeks later.

For microglia depletion, PLX3397 (Selleck, Houston, TX, USA) was dissolved in DMSO at 200 mg/ml and diluted to the working concentration. Eight weeks after AAV-*h*α-Syn injection, mice received intragastric feeding with 40 mg/kg of PLX3397 on 28 consecutive days. The control group received similar intragastric feeding with vehicle.

To detect the effects of AAV-Cntnap4 shRNA on A53T α-Syn mice, mice were administered AAV-GFP or AAV-Cntnap4 shRNA, and behavioral tests were performed 4 weeks later.

To examine the anti-inflammatory effects of C3aR antagonist, A53T α-Syn mice were injected with AAV-Cntnap4 shRNA for 4 weeks, and NPs@SB, CNPs@SB, and MCNPs@SB (equivalent dose of SB290157) were administered intravenously every other day for 19 days, before conducting behavioral tests.

### Behavioral tests

#### Open field test (OFT)

The procedure for OFT has been described previously [77]. Mice were placed in the center of a rectangular plastic box (40 × 40 × 40 cm). The movement of mice was recorded using a video tracking system (EthoVisione XT software, Beijing, China) for 15 min. The total distance, movement speed, and time spent by mice in the central zone were analyzed.

### Grasping test

The grasping test was performed according to the method reported in our previous study [77]. Mice were suspended on a horizontal metal wire of l mm diameter, placed 30 cm above the ground for 10 s using the two front paws. The grasping score was recorded as 3, 2, 1, and 0 if mice grasped the wire with two hind paws, mice grasped the wire with one hind paw, mice failed to grasp the wire, and mice fell, respectively.

### Pole-climbing test

The pole-climbing test was performed as described previously [77]. The test pole was set at a length of 75 cm and a width of 9 mm. Mice were placed on the top of the pole, and the time it took the mice to reach the ground from the top was recorded.

### Rotarod test

The rotarod test was performed as described previously [77]. Before the rotarod test, mice were placed on the Rotarod (Ugo Basile SRL, Gemonio, VA, Italy) at a speed of 10 rpm for training. Three days later, mice were placed on the rotarod cylinder that was accelerated from 4 to 40 rpm within 5 min. The latency time to falling of the animals was recorded.

### Y maze test

Mice were placed within the center zoom with three equal angles between the arms, with walls that were 30 cm long, 10 cm wide, and 20 cm high, for 8 min. The alternation score (%) and number of arm entries for each mouse was recorded. Non-overlapping entrance sequences were defined as spontaneous alternations.

### Elevated plus maze (EPM)

EPM consisted of two open arms (30 × 5 cm), two closed arms (30 × 5 × 15 cm), and a central zone (5 × 5 cm). Mice were placed in the central intersection and the total distance traveled, movement speed, open arm entries, and time spent in the open arm (%) were recorded using the video tracking system.

### RNA-sequencing (RNA-seq) and bioinformatics analysis

RNA-seq and bioinformatics analysis were performed similar to our previous study [75]. Briefly, RNA was isolated using Trizol (Invitrogen, Carlsbad, CA, USA), and cDNA libraries were prepared using the TruSeq Stranded mRNA LT Prep Kit (Illumina). Libraries were sequenced on a HiSeq 2500 instrument (Illumina) at the MGH Next Generation Sequencing Core Facility, using paired-end 50-bp sequencing. Sequencing reads were mapped by Novogene (Beijing, China). Read counts over transcripts were calculated using HTseq, followed by differential expression analysis using EdgeR. Genes were classified as differentially expressed based on the cutoffs of fold change (FC) > 1.6, false discovery rate (FDR) < 0.1, and *p* < 0.005. Kyoto Encyclopedia of Genes and Genomes (KEGG) analysis of differentially expressed genes (DEGs) were performed using the R package (v 3.5.1).

### Immunohistochemistry and immunofluorescence analyses

Mice brains were collected, post-fixed in 4% paraformaldehyde, and dehydrated in 20–30% sucrose solution. Brains were embedded in optimal cutting temperature (OCT) compound and cut into 15-μm serial coronal sections using a freezing microtome (Leica). Free-floating sections were blocked with 5% BSA and incubated with the primary antibody. In the immunohistochemistry assay, sections were incubated with biotin-conjugated antibody, followed by DAB staining using the UltraSensitive SP IHC Kit (MXB biotechnologies, China). Images were scanned under a microscope (Leica CS2, Hamburg, Germany). In the immunofluorescent assay, sections were incubated with fluorescent-labeled secondary antibody and images were acquired using a confocal microscope (SP8; Leica). Quantitative analysis was performed using the Image-Pro Plus 6.0 photogram analysis system (IPP 6.0, Media Cybernetics, Bethesda, MD, USA). The interaction of indicators, such as Iba1 and GFAP, was evaluated by quantifying the fluorescence signal intensity.

### Immunoblot analysis

Protein expression levels were examined using western blotting. Briefly, tissues were homogenized in RIPA buffer (Beyotime, Shanghai, China) and the supernatants were collected. Protein concentrations were quantified using the BCA Kit (Beyotime, Shanghai, China), and electrophoresis was performed using SDS-PAGE gels. Proteins were then transferred to polyvinylidene difluoride (PVDF) membranes. The membranes were blocked with 5% BSA and incubated at 4 °C overnight with primary antibodies, followed by HRP-conjugated secondary antibodies. The bands were visualized using enhanced chemiluminescence (ECL, Beyotime, Shanghai, China). Images were captured using the GeneGnome XRQ Chemiluminescence imaging system (Gene Company, Hong Kong, China). ImageJ software was used to analyze the optical density of bands.

### qRT-PCR

Total RNA was isolated using the Trizol reagent (Invitrogen, Carlsbad, CA, USA), and RNA quantity was assessed using Nanodrop (Agilent Technologies, California, USA). Then, cDNA was generated from 1 μg of total RNA per sample using the cDNA Reverse Transcription Kit (QIAGEN, Waltham, MA, USA). Quantitative PCR was performed using the primers listed in Table S1. Each sample was compared to GAPDH as the internal control. Data were recorded from three separate experiments, with each performed in triplicate.

### Transmission electron microscopy (TEM)

The ultrastructural morphologies of nigral synaptic vesicles, mitochondria, and autolysosome were analyzed using TEM, similar to our previous study [77]. After fixing and dehydration with different concentrations of ethanol and acetone, SN tissues were embedded with 812 embedding agents (SPI-Pon 812 Epoxy Resin Monomer; SPI, Shanxi, China). Following polymerization, sections were cut using a Leica EM UC7 (Leica Microsys, Germany) and placed on copper grids. The grids were post-stained with uranyl acetate and bismuth subnitrate. The sections were observed using TEM (HT7700, Hitachi, Tokyo, Japan).

### Enzyme-linked immunosorbent assay (ELISA)

ELISA was performed as described previously [77]. Nigral IL-1β, IL-6, and TNF-α levels were measured using ELISA kits (Shanghai Enzyme-linked Biotechnology, Shanghai, China) according to the manufacturer’s instructions. OD values were measured using a Multiscan Spectrum (PerkinElmer, MA, USA) at 450 nm, and the results are expressed as pg per mg protein (pg mg^−1^ protein).

### Synthesis of DSPE-PEG-CRT and DSPE-PEG-MG1 macromolecule

Briefly, 20 mg DSPE-PEG-NHS and 9.35 mg CRT or 8.83 mg MG1 were dissolved in 10 mL of N, N dimethyl, before adding 20 μL of 1, 3-malonediamine and stirring gently at room temperature for 48 h. The resulting reaction solution was added into the dialysis membrane bag (cut-off molecular weight of 500 Da) and dialysis was performed with distilled water for 48 h. The water was changed every 6 h and the solution was freeze-dried. Finally, the DSPE-PEG-CRT and DSPE-PEG- MG1 powder were stored at −20 °C and evaluated using a Fourier Transform Infrared Spectrometer.

### In vivo imaging and biodistribution analysis

Mice were intravenously injected with Cy5.5-labeled NPs@SB, CNPs@SB, and MCNPs@SB (200 μL, containing 2 mg/mL SB290157 and 20 μg/mL Cy5.5). The fluorescence signals of Cy5.5 were obtained and semiquantitatively analyzed using an ex/in vivo IVIS imaging system (IVIS Spectrum, PerkinElmer, Waltham, MA, USA) (Excitation wavelength: 674 nm; Emission wavelength: 692 nm). After 24 h, mice were euthanized, and the organs (heart, liver, spleen, lung, kidneys, and brain) were collected for imaging using the *ex/*in vivo IVIS imaging system (PerkinElmer, Waltham, MA, USA).

### Statistics

Data are presented as the mean ± standard error of the mean (SEM). Data were analyzed using Student’s *t*-test, one-way ANOVA followed by Tukey’s post hoc test, or two-way ANOVA followed by Bonferroni’s multiple comparisons test, as appropriate. Differences with a *p*-value < 0.05 were considered statistically significant. The statistical analyses were performed using GraphPad Prism 9.0 (GraphPad Software, La Jolla, CA, USA). *P*-values are represented as *p < 0.05 and **p < 0.01.

## Supplementary information

Supplementary Information

Supplementary Table 1

Supplementary Figure 1

Supplementary Figure 2

Supplementary Figure 3

Supplementary Figure 4

Supplementary Figure 5

Supplementary Figure 6

Supplementary Figure 7

Supplementary Figure 8

Supplementary Figure 9

Supplementary Figure 10

Supplementary Figure 11

Supplementary Figure 12

Supplementary Figure 13

Supplementary Figure 14

Supplementary Figure 15

Supplementary Figure 16

Supplementary Figure 17

Supplementary Figure 18

Reproducibility checklist

Raw images for western blots

## Data Availability

The datasets used and analyzed during the current study are available from the corresponding author upon reasonable request.
