# Supplementary material for: Cntnap4 partial deficiency exacerbates α-synuclein pathology through astrocyte–microglia C3-C3aR pathway
Source: Cell Death Dis. 2023 Apr 22;14(4):285. doi: 10.1038/s41419-023-05807-y (PMC10122675; doi:10.1038/s41419-023-05807-y)
Supplement: Supplementary file 1 — Supplementary Information [file 41419_2023_5807_MOESM1_ESM.docx]

**Supplementary Information**

***Cntnap4* partial deficiency exacerbates α-synuclein pathology through astrocyte–microglia C3-C3aR pathway**

**Supporting Methods**

**Reagents**

Anti-Cntnap4 (bs-11076R-2) and anti-Cntnap4 (orb544737) antibodies were purchased from Bioss (Beijing, China) and Biorbyt LLC (San Francisco, CA, USA), respectively. Anti-human α-synuclein (ab138501), phospho-α-synuclein (Ser129) (ab51253), CD68 (ab53444), and C1q (ab71940) antibodies were purchased from Abcam (Cambridge, MA, USA). Anti-mouse α-synuclein (610786) antibody was purchased from BD Biosciences (Franklin Lakes, NJ, USA). Anti-glial fibrillary acidic protein (GFAP; MAB360) antibody was purchased from Millipore (Billerica, MA, USA). Anti-ionized calcium binding adapter molecule (Iba1) (019–19741) antibody was purchased from FUJIFILM Wako (Osaka, Japan). anti-FTH1 (#4393), GPX4 (#52455), anti-synapsin Ⅰ (#5297), anti-syntaxin (#18572), anti-SNAP25 (#5308), and anti-PSD-95 (#3450) antibodies were purchased from Cell Signaling Technology (Danvers, MA, USA). Anti-synapsin Ⅲ (106 303) antibody was purchased from Synaptic Systems (Goettingen, Germany). Anti-TH (F-11, sc-25269), anti-C3 (sc-28294), anti-C3aR (sc-74522), and anti-NCOA4 (sc-373739) antibodies were purchased from Santa Cruz Biotechnology (Dallas, TX, USA). Anti-GAPDH (60004–1) antibody was purchased from Proteintech Group (Rosemont, IL, USA). DyLight 488 goat anti-mouse IgG (H + L) (70- GAM4882) and DyLight 594 goat anti-rabbit IgG (H + L) (70-GAR5942) antibodies were purchased from Multi Sciences (Hangzhou, China). Horseradish peroxidase (HRP)-labeled goat anti-rabbit IgG and HRP-labeled goat anti-mouse IgG were purchased from Beyotime Biotechnology (Shanghai, China). Cyclic peptide CRT (sequence: CRTIGPSVC) peptide and MG1 (sequence: CHHSSSAR) peptide were synthesized by Sangon Biotech Co. Ltd. (Shanghai, China); and 1,2-dioleoyl-sn-glycero-3-phosphoethanolamine (DOPE, Mn = 744), 1,2-dioleoyl-3-trimethylammonium propane (DOTAP) and DSPE-PEG2000-NHS (Mn = 2000 Da) were purchased from Xi’an ruixi Biological Technology Co. Ltd. (Xi’an, China).

**Measurement of cytokine secretion**

Serum cytokine levels were examined using the customized mouse Magnetic Luminex assay kit (Bio-Techne, Minneapolis, MN, USA) according to the manufacturer’s instructions. The measurements were performed using a Bio-Rad BioPlex 200 system (Bio-Rad Laboratories, Basel, Switzerland) and data were analyzed using Bio-Plex version 6.0 (Bio-Rad Laboratories).

**Synthesis of nanoparticles**

Initially, 80 mg DOPAT, 17.04 mg DOPE, 5.1 mg DSPE-PEG-CRT, or 3.4 mg DSPE-PEG-MG1 were dissolved in 10 mL of CHCl_3_. Subsequently, the solutions were evaporated by decompressed rotary evaporation and a thin film was formed on the wall of a 50-mL round bottom flask. The residual solvents in the film were removed by vacuum freeze-drying, and then 2 mL of ultrapure water was added to disperse the dry product. After ultrasonication for 5 min in ice-bath, the liposomes of MG1-CRT-SBNPs were obtained. NPs, NPs@SB, and CNPs@SB were prepared similarly. Finally, the DSPE-PEG-CRT and DSPE-PEG-MG1 were characterized by Fourier transform infrared spectrometer.

**Characterization of nanoparticles**

The size distribution, PDI, and ζ-potential of NPs, NPs@SB, CNPs@SB, and MCNPs@SB were investigated by Zetasizer Nano ZS (Malvern, U.K.). The particle morphology was captured by transmission electron microscopy (FEI Tecnai G2 F20 S-Twin, USA). The absorption spectra were obtained by UV–vis spectrometry (Lambda 35, PerkinElmer, Waltham, MA, USA).

**Encapsulation efficiency and drug loading**

The encapsulation efficiencies of NPs@SB, CNPs@SB, and MCNPs@SB were determined by ultrafiltration. SB290157 was dissolved in aqueous solution with 0.25% sodium dodecyl sulfate. NPs@SB, CNPs@SB, and MCNPs@SB were separated by ultracentrifugation (Millipore, Billerica, MA, USA). NPs@SB, CNPs@SB, and MCNPs@SB were retained and free SB290157 was separated from the filtrate through ultracentrifugation. Finally, the content of SB290157 in NPs@SB, CNPs@SB, and MCNPs@SB were determined by UV–vis spectrometry, and the encapsulation efficiency was calculated as follows:

EE% = (Wt – Wf)/Wt × 100%,

where Wt refers to the initial concentration of the SB290157 to the NPs@SB, CNPs@SB, and MCNPs@SB, and Wf refers to the free SB290157 in the filtrate.

Next, collected samples of NPs@SB, CNPs@SB, and MCNPs@SB were lyophilized. Freeze-dried powders of NPs@SB, CNPs@SB, and MCNPs@SB were dissolved thoroughly in ddH_2_O, before determination by UV/vis spectrometry. The loading efficiency was calculated as follows:

LE% = W_F_/W_N_ × 100%,

where W_F_ refers to the weight of the SB290157 in NPs@SB, CNPs@SB, and MCNPs@SB, and W_N_ refers to the weight of NPs@SB, CNPs@SB, and MCNPs@SB, respectively.

**Release profiles of free SB290157 and MCNPs@SB**

Briefly, SB290157 and MCNPs@SB (containing 2 mg/mL SB290157) were suspended in 10 mL of PBS (pH 7.4) in a dialysis bag with a molecular weight cutoff of 100 kDa. The solution was dialyzed against 10 mL of PBS (pH 7.4), with 0.25% sodium dodecyl sulfate as a dissolution medium, under 200 rpm stirring at 37°C. At different time points, PBS was collected and the absorbance of resulting SB290157 was measured at 270 nm. The concentration of SB290157 in solution was determined using a standard curve of SB290157 in PBS.

**Supporting Figure Legends**

**Figure S1. Effect of partial *Cntnap4* partial deficiency on the striatal α-synuclein pathology and nigral DA neuron expression in AAV-*h*α-Syn injected mice.** (A) Immunohistochemical staining of striatal TH-positive cells in WT and Cntnap4^+/-^ mice injected with either AAV-GFP or AAV-*h*α-Syn. Scale bars: 1 mm. (B) Quantification of striatal TH-positive density; n = 6 per group. (C and D) The protein expression levels of *h*α-Syn and mouse α-synuclein were determined using western blotting; n = 3 per group. Results are expressed as the mean ± SEM. ^**^*p* < 0.01, ^*^*p* < 0.05 vs. WT; ^#^*p* < 0.05 vs. AAV-*h*α-Syn. Statistical significance was determined using two-way ANOVA + Bonferroni’s multiple comparisons test.

**Figure S2. Effect of α-synuclein burden on the nigral synaptic protein expression in *Cntnap4* partial deficiency mice.** (A–C) The nigral protein expression levels of synapsin I, synapsin III, syntaxin, SNAP25, and PSD-95 in WT and Cntnap4^+/-^ mice injected with either AAV-GFP or AAV-*h*α-Syn were determined using western blotting; n = 3 per group. Results are expressed as the mean ± SEM. ^**^*p* < 0.01, ^*^*p* < 0.05 vs. WT. Statistical significance was determined using two-way ANOVA + Bonferroni’s multiple comparisons test.

**Figure S3. Behavioral tests of *Cntnap4* partially deficient mice by α-synuclein burden.** (A and B) The number of entries to the center zone and duration in the center in the open-field of WT and Cntnap4^+/-^ mice injected with either AAV-GFP or AAV-*h*α-Syn. (C) The grasping test was used to examine the grip strength of mice. (D and E) Y maze test of alternative behavior and number of arm entries. (F–I) EPM test of total distance travelled, open arm entries, time spent in the open arm, and movement speed; n = 11, 11, 10, and 7 in the WT + AAV-GFP, WT + AAV-*h*α-Syn, Cntnap4^+/-^ + AAV-GFP, and Cntnap4^+/-^ + AAV-*h*α-Syn groups, respectively. Results are expressed as the mean ± SEM. ^**^*p* < 0.01, ^*^*p* < 0.05 vs. WT; ^##^*p* < 0.01 vs. AAV-*h*α-Syn. Statistical significance was determined using two-way ANOVA + Bonferroni’s multiple comparisons test.

**Figure S4. Pathways enriched by upregulated DEGs between Cntnap4^+/-^ + AAV-*h*α-Syn and Cntnap4^+/-^ mice.** (A) PCA score plots revealed a distinct separation of components in WT and Cntnap4^+/-^ mice injected with either AAV-GFP or AAV-*h*α-Syn. (B) DEGs between the Cntnap4^+/-^ + AAV-*h*α-Syn and Cntnap4^+/-^ groups are shown in a volcano plot. (C and D) The representative KEGG pathways enriched by upregulated DEGs between Cntnap4^+/-^ + AAV-*h*α-Syn and Cntnap4^+/-^ group are shown.

**Figure S5. Pathways enriched by upregulated DEGs between Cntnap4^+/-^ + AAV-*h*α-Syn and AAV-*h*α-Syn mice.** (A) DEGs between the Cntnap4^+/-^ + AAV-*h*α-Syn and AAV-*h*α-Syn groups are shown in a volcano plot. (B) The representative KEGG pathways enriched by upregulated DEGs between Cntnap4^+/-^ + AAV-*h*α-Syn and AAV-*h*α-Syn group are shown.

**Figure S6. Effect of α-synuclein burden on the serum cytokine expression in *Cntnap4* partially deficient mice.** (A–L) Serum expression levels of IL-6, G-CSF, IL-3, IL-2, TNF-α, IL-5, IL-21 p40, IL-21 p70, IL-1α, IL-10, IL-17A, and IFN-γ were determined using Mouse Magnetic Luminex Assay; n = 8, 8, 8, and 6 in the WT + AAV-GFP, WT + AAV-*h*α-Syn, Cntnap4^+/-^ + AAV-GFP, and Cntnap4^+/-^ + AAV-*h*α-Syn groups, respectively. Results are expressed as the mean ± SEM. ^**^*p* < 0.01, ^*^*p* < 0.05 vs. WT. Statistical significance was determined using two-way ANOVA + Bonferroni’s multiple comparisons test.

**Figure S7. Serum cytokine expression levels by α-synuclein burden in Cntnap4^+/-^ mice.** (A–J) Serum expression levels of IL-4, IL-9, MCP-1, IL-13, GM-CSF, Eotaxin, MIP-1α, MIP-1β, RANTES, and KC were determined using Mouse Magnetic Luminex Assay; n = 8, 8, 8, and 6 in the WT + AAV-GFP, WT + AAV-*h*α-Syn, Cntnap4^+/-^ + AAV-GFP, and Cntnap4^+/-^ + AAV-*h*α-Syn groups, respectively. Results are expressed as the mean ± SEM. Statistical significance was determined using two-way ANOVA + Bonferroni’s multiple comparisons test.

**Figure S8. Microglial expression upon α-synuclein burden in Cntnap4^+/-^ mice.** (A) Co-staining of Iba1 with TH in the SNpc of WT and Cntnap4^+/-^ mice injected with either AAV-GFP or AAV-*h*α-Syn. (B) Quantification of the interaction area of Iba1 and TH cells. Scale bars: 40 μm. Magnified images are shown in the right column of the panel. Scale bars: 8 μm. (C) Co-staining of *h*α-Syn with C1q in the SNpc of WT and Cntnap4^+/-^ mice injected with either AAV-GFP or AAV-*h*α-Syn. (D) Quantification of the interaction area of *h*α-Syn with C1q cells. Scale bars: 40 μm. Magnified images are shown in the right column of the panel. Scale bars: 8 μm; n = 5–8. Results are expressed as the mean ± SEM. ^*^*p* < 0.05 vs. WT; ^#^*p* < 0.05 vs. AAV-*h*α-Syn; ^&^*p* < 0.05 vs. Cntnap4^+/-^. Statistical significance was determined using two-way ANOVA + Bonferroni’s multiple comparisons test.

**Figure S9. Pathways enriched by downregulated DEGs between Cntnap4^+/-^ + AAV-*h*α-Syn and Cntnap4^+/-^ or AAV-*h*α-Syn mice.** (A and B) The representative KEGG pathways enriched by downregulated DEGs between the Cntnap4^+/-^ + AAV-*h*α-Syn and Cntnap4^+/-^ groups are shown. (C) The downregulated DEGs enriched in the “Dopaminergic synapse” and “Synaptic vesicle cycle.” (D) The representative KEGG pathways enriched by downregulated DEGs between Cntnap4^+/-^ + AAV-*h*α-Syn and AAV-*h*α-Syn group are shown.

**Figure S10. Overexpression of α-synuclein hampers synaptic plasticity in Cntnap4^+/-^ mice.** Ultrastructural analysis and quantification of synaptic vesicles in the SNpc of WT and Cntnap4^+/-^ mice injected with either AAV-GFP or AAV-*h*α-Syn. Scale bars: 500 nm; n = 10 per group. Results are expressed as the mean ± SEM. ^**^*p* < 0.01 vs. WT. Statistical significance was determined using two-way ANOVA + Bonferroni’s multiple comparisons test.

**Figure S11. *C3* mRNA expression levels.** (A) *C3* mRNA expression in astrocytes treated with the cultured supernatant from MN9D cells (NC siRNA + PBS, NC siRNA + *h*α-Syn, Cntnap4 siRNA + PBS, and Cntnap4 siRNA + *h*α-Syn) at 3, 6, 12 and 24 h; n = 3 per group. (B) Astrocytes were treated with C3 siRNA sequences 1–3 for 48 h, and then the *C3* mRNA expression level was determined by qRT-PCR; n = 3 per group. We chose C3 siRNA-1 for the following experiments. Results are expressed as the mean ± SEM. ^**^*p* < 0.01, ^*^*p* < 0.05 vs. NC siRNA; ^#^*p* < 0.05 vs. *h*α-Syn; ^&^*p* < 0.05 vs. Cntnap4 siRNA. Statistical significance was determined using one-way ANOVA + Tukey’s multiple comparisons test.

**Figure S12. C3aR expression levels in primary microglia.** Microglia were treated with *h*α-Syn for 24 h. Then, the mRNA expression (A) and protein expression (B and C) of C3aR were examined by qRT-PCR and western blotting. Microglia were treated with cultured supernatant from MN9D cells (NC siRNA + PBS, NC siRNA + *h*α-Syn, Cntnap4 siRNA + PBS, and Cntnap4 siRNA + *h*α-Syn) for 24 h. Then, the mRNA expression (D) and protein expression (E and F) of C3aR were examined by qRT-PCR and western blotting; n = 3 per group. Results are expressed as the mean ± SEM. Statistical significance was determined using Student’s *t*-test (A and C), one-way ANOVA + Tukey’s multiple comparisons test (D and F).

**Figure S13. PLX3397 interrupts astrocyte–microglial C3-C3aR signaling in Cntnap4^+/-^ mice injected AAV-*h*α-Syn.** Quantification of GFAP-merging Iba1 cells (A) and GFAP-merging C3 cells (B) in the SNpc of WT, Cntnap4^+/-^ + AAV-*h*α-Syn, and Cntnap4^+/-^ + AAV-*h*α-Syn + PLX3397 groups; n = 7–11. Results are expressed as the mean ± SEM. ^**^*p* < 0.01 vs. WT; ^##^*p* < 0.01 vs. Cntnap4^+/-^ + AAV-*h*α-Syn. Statistical significance was determined using one-way ANOVA + Tukey’s multiple comparisons test.

**Figure S14. PLX3397 reduces the pro-inflammatory response and nigral neuron death in AAV-*h*α-Syn mice.** (A and B) The mRNA expression levels of *Il-1b*, *Il-6*, *Tnfa*, *Ifng*, *Csf1r*, *Cx3cr1*, *Tmem119*, and *P2ry12* were determined by qRT-PCR; n = 3 per group. (C) Immunohistochemical staining and quantification of TH-positive cells in WT, AAV-*h*α-Syn, and AAV-*h*α-Syn + PLX3397 groups. Scale bars: 1mm, 200 μm. Magnified images of TH-positive cells in the SNpc are shown in the right column. Scale bars: 50 μm; n = 6–8. Results are expressed as the mean ± SEM. ^**^*p* < 0.01, ^*^*p* < 0.05 vs. WT; ^##^*p* < 0.01 vs. AAV-*h*α-Syn. Statistical significance was determined using one-way ANOVA + Tukey’s multiple comparisons test.

**Figure S15. Infrared absorption spectra of DSPE-PEG-NHS, CRT, and DSPE-PEG-CRT.** (A) DSPE-PEG-NHS infrared absorption spectra. (B) Infrared absorption spectrum of polypeptide CRT. (C) Infrared absorption spectrum of DSPE-PEG-CRT. (D) Infrared absorption spectra of DSPE-PEG-NHS, CRT, and DSPE-PEG-CRT.

**Figure S16. Infrared absorption spectra of DSPE-PEG-NHS, MG1, and DSPE-PEG-MG1.** (A) Infrared absorption spectra of DSPE-PEG-NHS. (B) Infrared absorption spectrum of polypeptide MG1. (C) Infrared absorption spectra of DSPE-PEG-MG1. (D) Infrared absorption spectra of DSPE-PEG-NHS, MG1, and DSPE-PEG-MG1.

**Figure S17. Parameters of nanoparticles.** (A) Polydispersity index (PDI) analysis of NPs, NPs@SB, CNPs@SB, and MCNPs@SB; n = 3 per group. (B) The Z-average size of NPs, NPs@SB, CNPs@SB and MCNPs@SB; n = 3 per group. (C) UV–vis absorbance of SB. (D) The standard curves were linear over the range of 62.5–500 μg/mL for SB. (E) UV–vis absorbance of SB, NPs@SB, CNPs@SB, and MCNPs@SB. Results are expressed as the mean ± SEM.

**Figure S18. Quantification of Iba1 with C3aR in the SNpc of WT, shPD, shPD-NPs@SB, shPD-CNPs@SB, and shPD-MCNPs@SB.** Results are expressed as the mean ± SEM; n = 6–7. ^**^*p* < 0.01 vs. WT; ^#^*p* < 0.05 vs. shPD. Statistical significance was determined using one-way ANOVA + Tukey’s multiple comparisons test.
