# Supplementary material for: Cntnap4 partial deficiency exacerbates α-synuclein pathology through astrocyte–microglia C3-C3aR pathway
Source: Cell Death Dis. 2023 Apr 22;14(4):285. doi: 10.1038/s41419-023-05807-y (PMC10122675; doi:10.1038/s41419-023-05807-y)
Supplement: Supplementary file 2 — Supplementary Table 1 [file 41419_2023_5807_MOESM2_ESM.docx]

**Table S1. Primer sequences used for qRT-PCR**

| Mouse genes | Primer sequence (5′-3′) |
| --- | --- |
| *C1qa* | F: GGACTGGTATCCGAGGTTTTAA  R: GATATTGCCTGGATTGCCTTTC |
| *C1qb* | F: GCTAGAGCAAGAGGAGGTTGTTCAC  R: TCAGGGAAAAGCAGAAAGCCAGTG |
| *C1qc* | F: TACTTCGTCTACTACACATCGC  R: GGAAACAGTAGGAAACCAGAGA |
| *C1s1* | F: GCATTGTCTTTCAAACTGACCT  R: ACACAGGTTATCTTCACCACAT |
| *C3* | F: GTTTCTGAACACAGCCAAAGAT  R: GACATCTGTTTGATATTGGGCC |
| *C4b* | F: GAGTCCAGAGTTCAGTACACTG  R: CAGTCTCAAAGTGACTCACGTA |
| *Il-1b* | F: AATGCCACCTTTTGACAGTGAT  R: TGCTGCGAGATTTGAAGCTG |
| *Il-6* | F: AGGATACCACTCCCAACAGACC  R: AAGTGCATCATCGTTCATACA |
| *Tnfa* | F: CACGTCGTAGCAAACCACC  R: TGAGATCCATGCCGTTGGC |
| *Ifng* | F: TGGCAGGAGATGTCTACACT  R: GAAGCACCAGGTGTCAAGTC |
| *Tgfb* | F: ATTCCTGGCGTTACCTTGG  R: AGCCCTGTATTCCGTCTCCT |
| *Csf1r* | F: CCTCAAACGTGGAGACACCAA  R: CGTGTGCCAACATCATTGCT |
| *Cx3cr1* | F: CAACCCCTTTATCTACGCCTT  R: GACCCATCTCCCTCGCTTG |
| *Tmem119* | F: CTGACATTCTGGCTGCTACC  R: CACCCTTCACAGGCTTTGCTC |
| *P2ry12* | F: TTTGCTGGGCTCATCACGAAC  R: ACTGAAGTAACTTGGCACACC |
| *Gapdh* | F: ACGGGAAGCTCACTGGCATGGCCTT  R: CATGAGGTCCACCACCCTGTTGCTG |

The primer sequences of *C1qa*, *C1qb*, *C1qc*, *C1s1*, *C3*, *C4b*, *Il-1b*, *Il-6*, *Tnfa*, *Ifng*, *Tgfb*, *Csf1r*, *Cx3cr1*, *Tmem119*, *P2ry12*, and *Gapdh*.
