# Supplementary figures and images for: Cntnap4 partial deficiency exacerbates α-synuclein pathology through astrocyte–microglia C3-C3aR pathway
Source: Cell Death Dis. 2023 Apr 22;14(4):285. doi: 10.1038/s41419-023-05807-y (PMC10122675; doi:10.1038/s41419-023-05807-y)

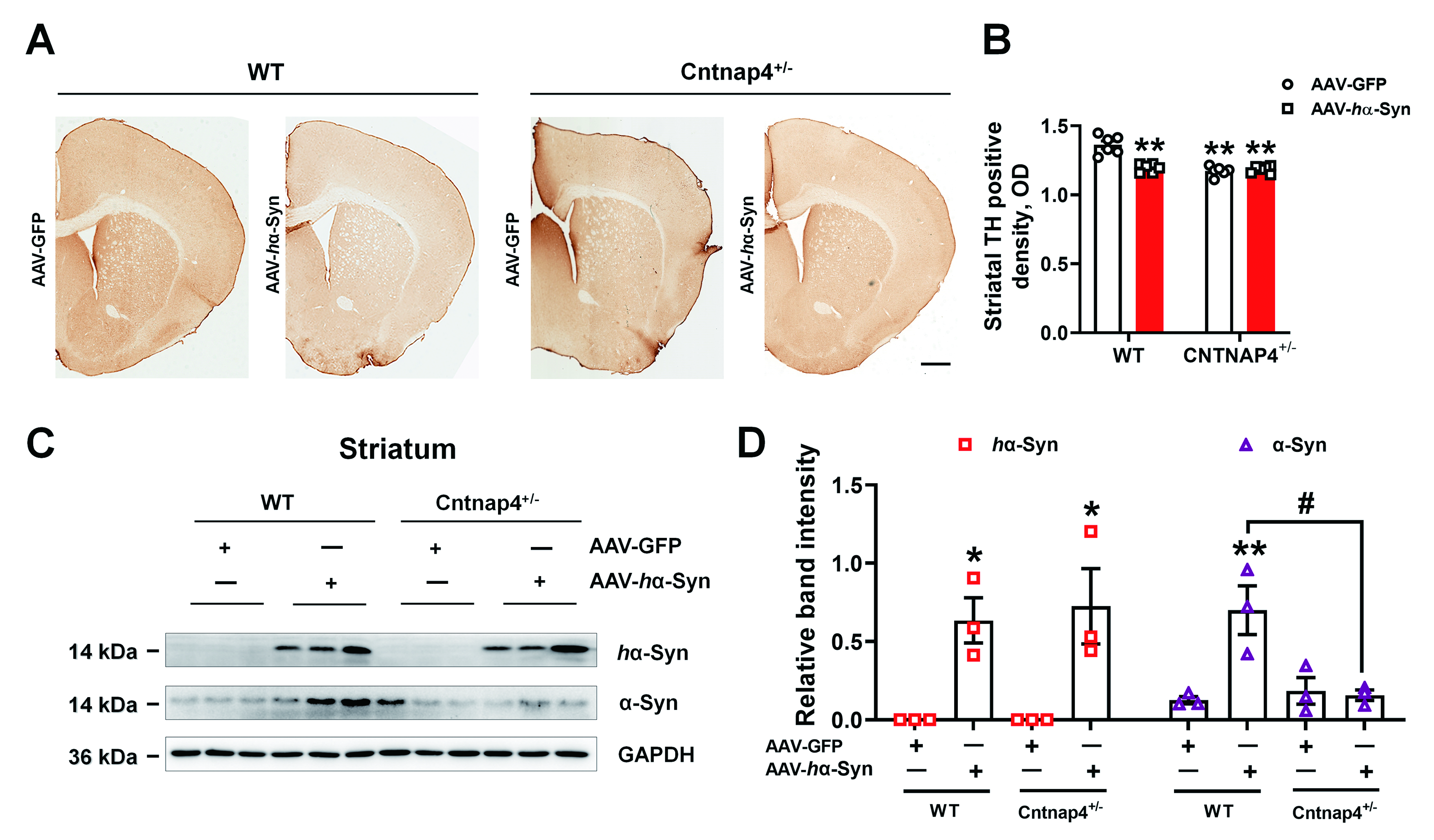

Supplement: Supplementary file 3 — Supplementary Figure 1 [file 41419_2023_5807_MOESM3_ESM.tif]

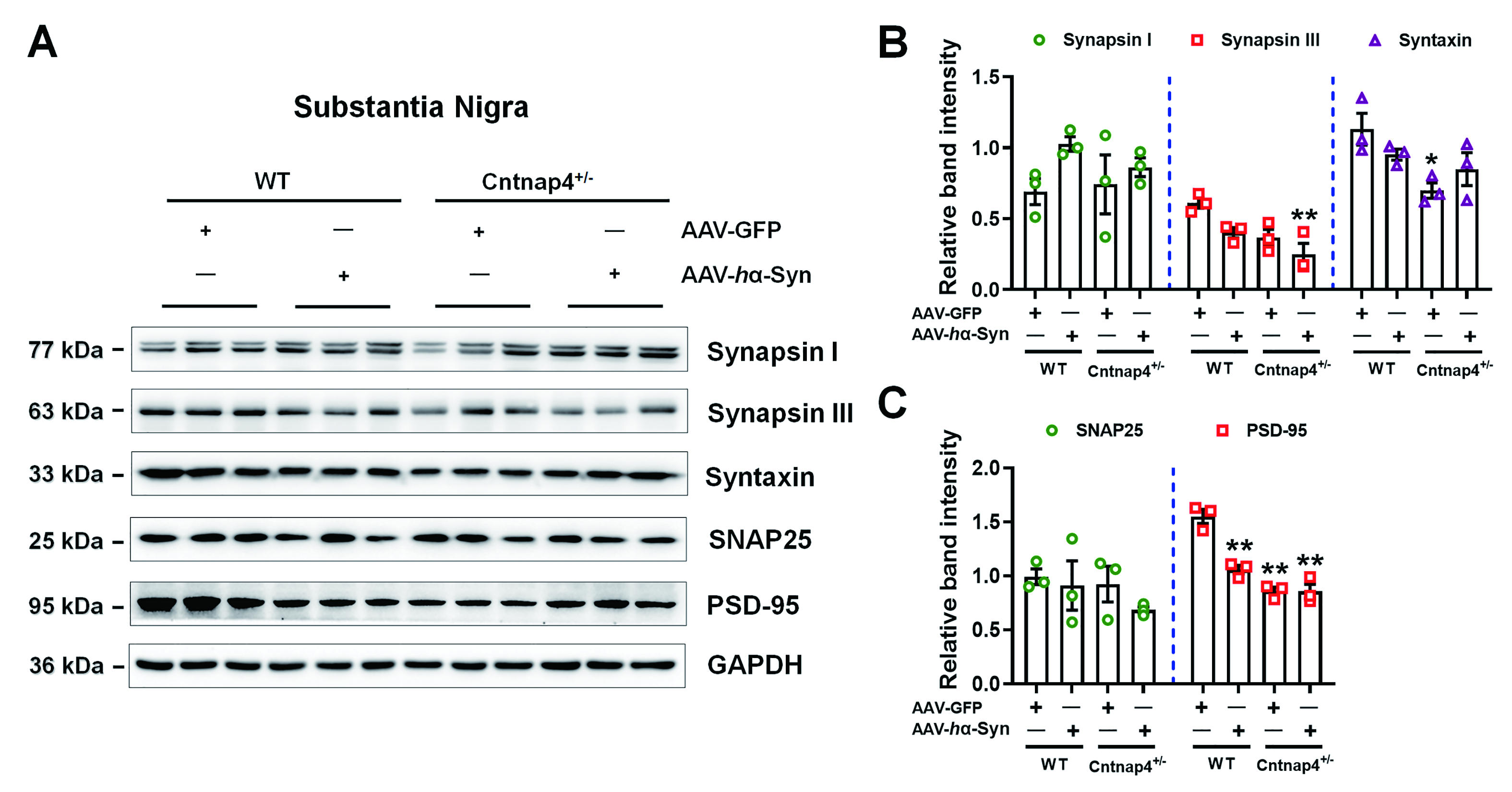

Supplement: Supplementary file 4 — Supplementary Figure 2 [file 41419_2023_5807_MOESM4_ESM.tif]

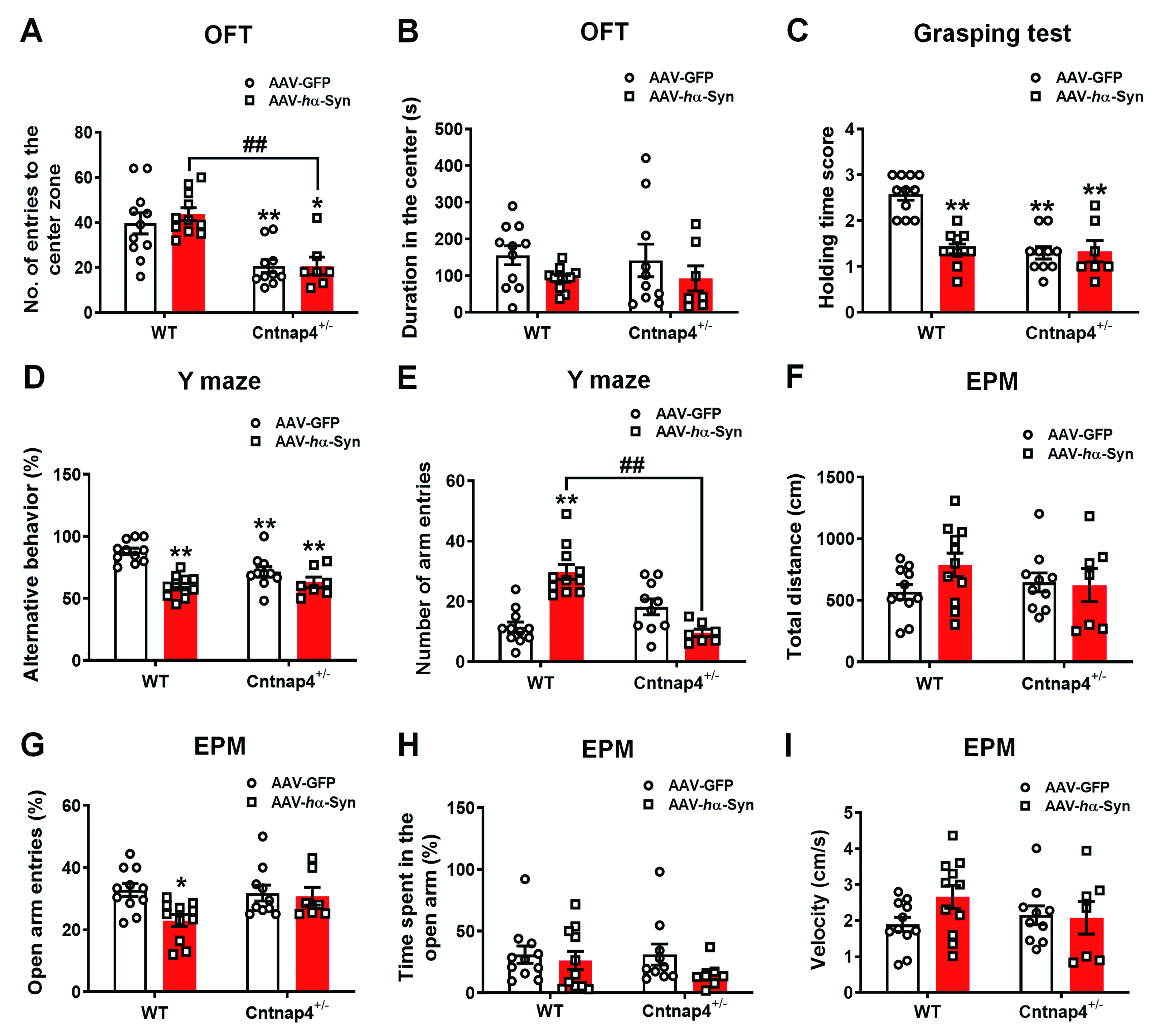

Supplement: Supplementary file 5 — Supplementary Figure 3 [file 41419_2023_5807_MOESM5_ESM.tif]

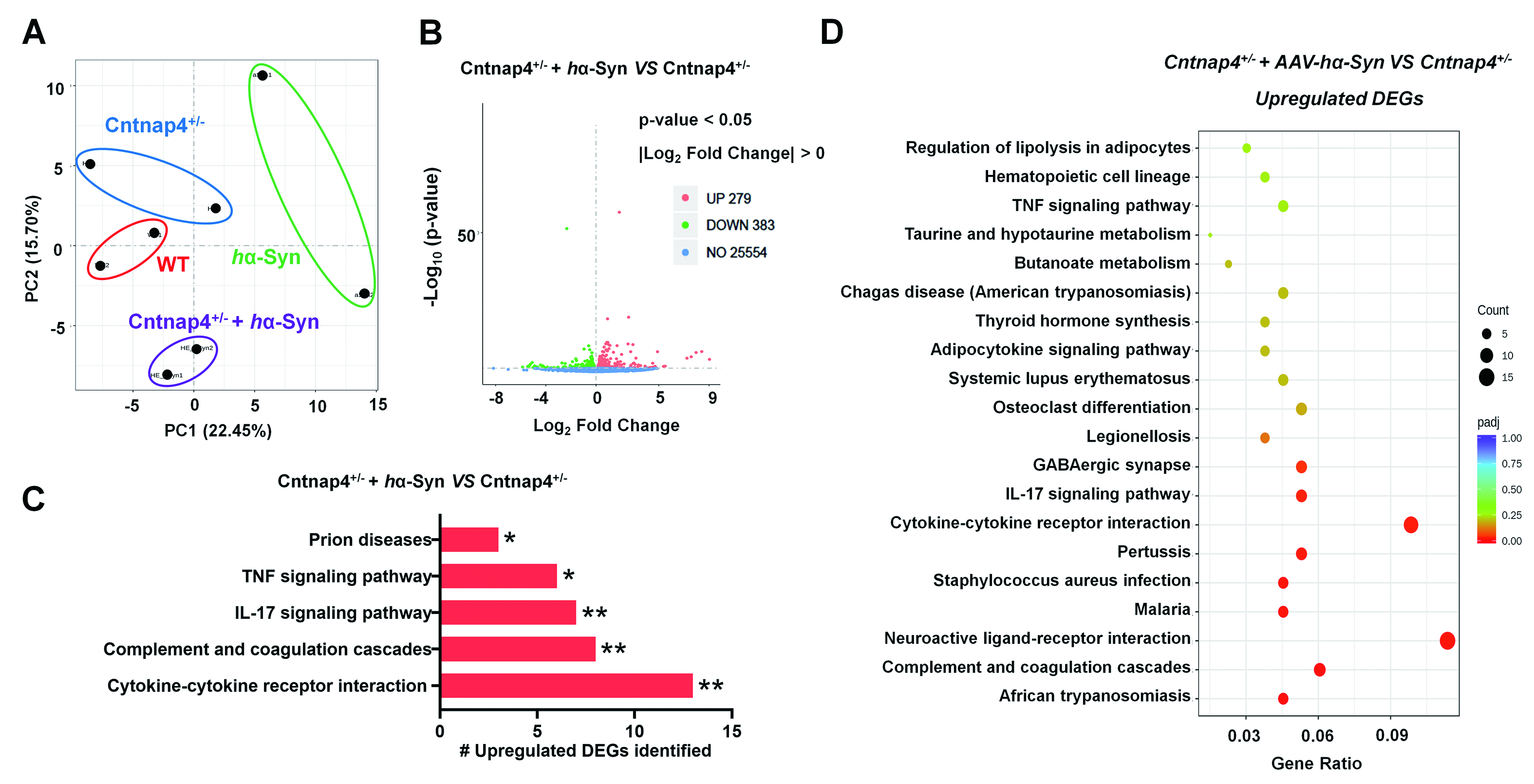

Supplement: Supplementary file 6 — Supplementary Figure 4 [file 41419_2023_5807_MOESM6_ESM.tif]

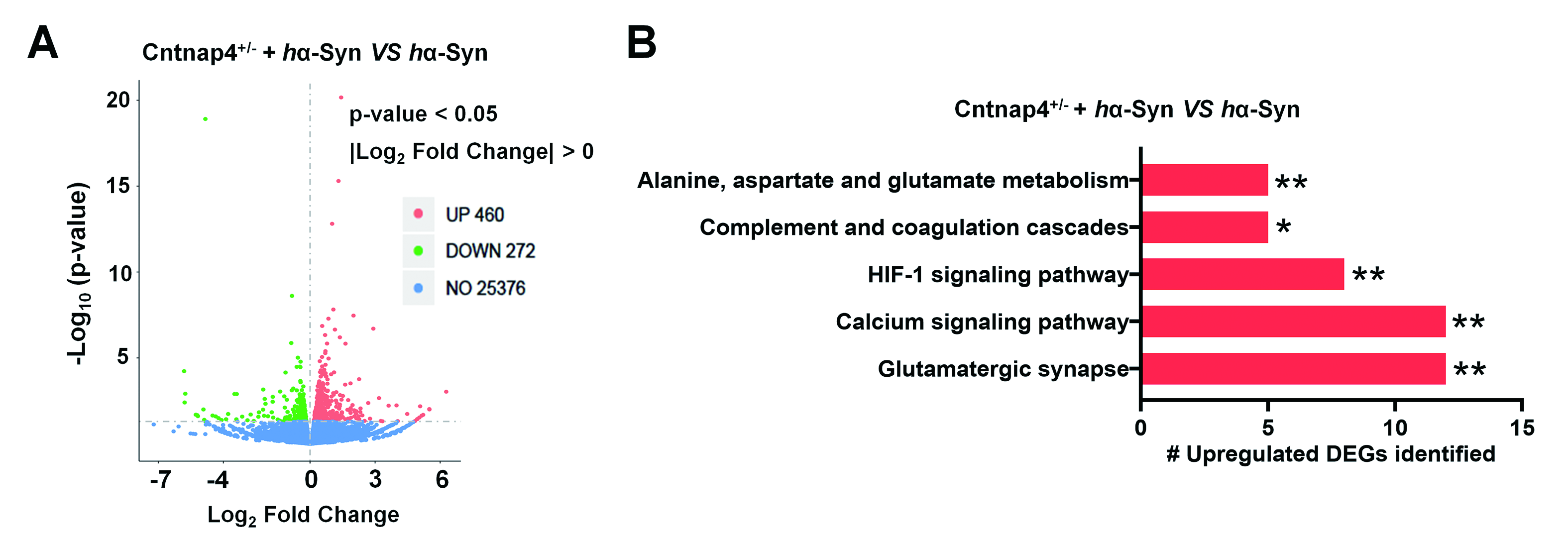

Supplement: Supplementary file 7 — Supplementary Figure 5 [file 41419_2023_5807_MOESM7_ESM.tif]

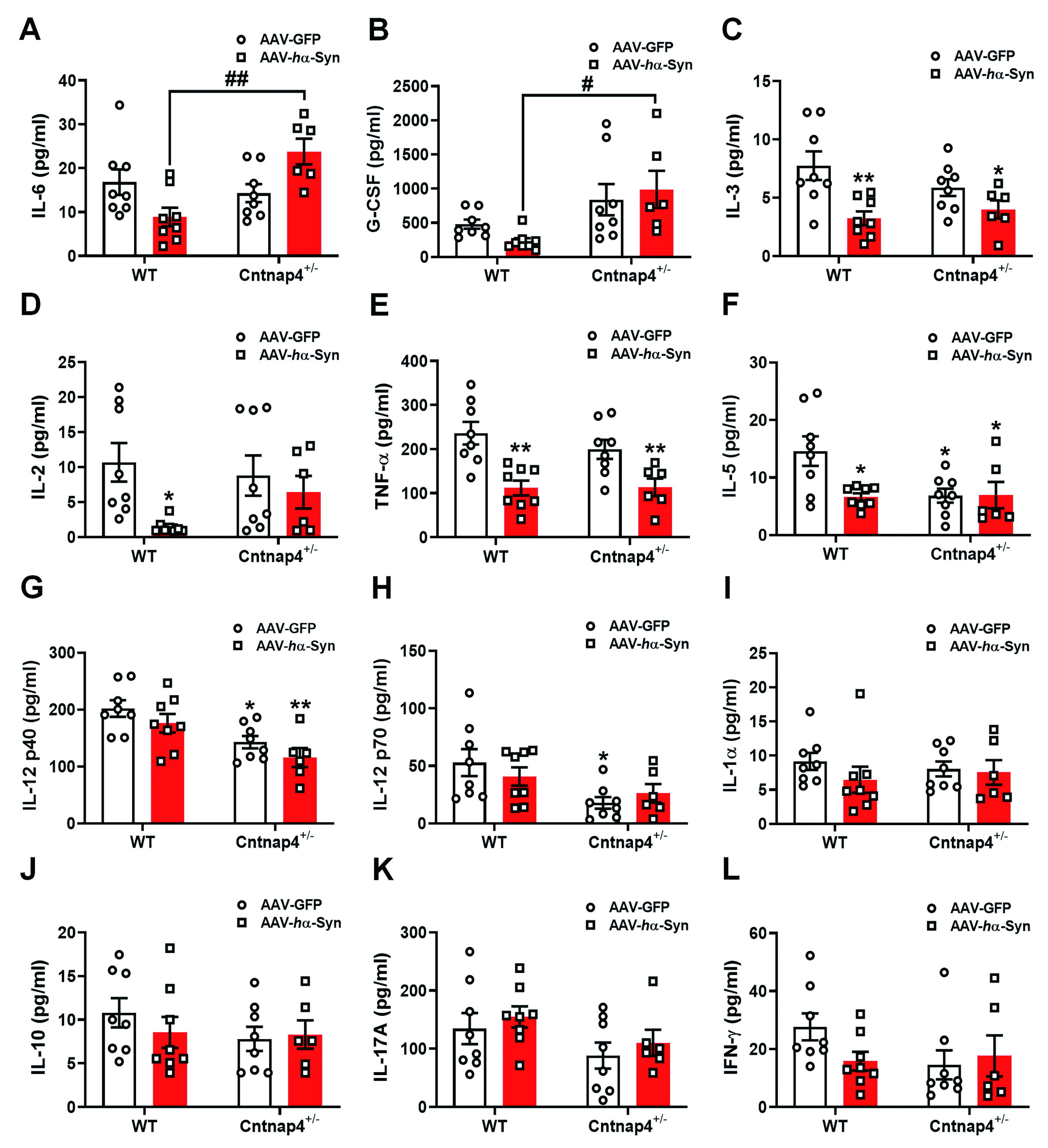

Supplement: Supplementary file 8 — Supplementary Figure 6 [file 41419_2023_5807_MOESM8_ESM.tif]

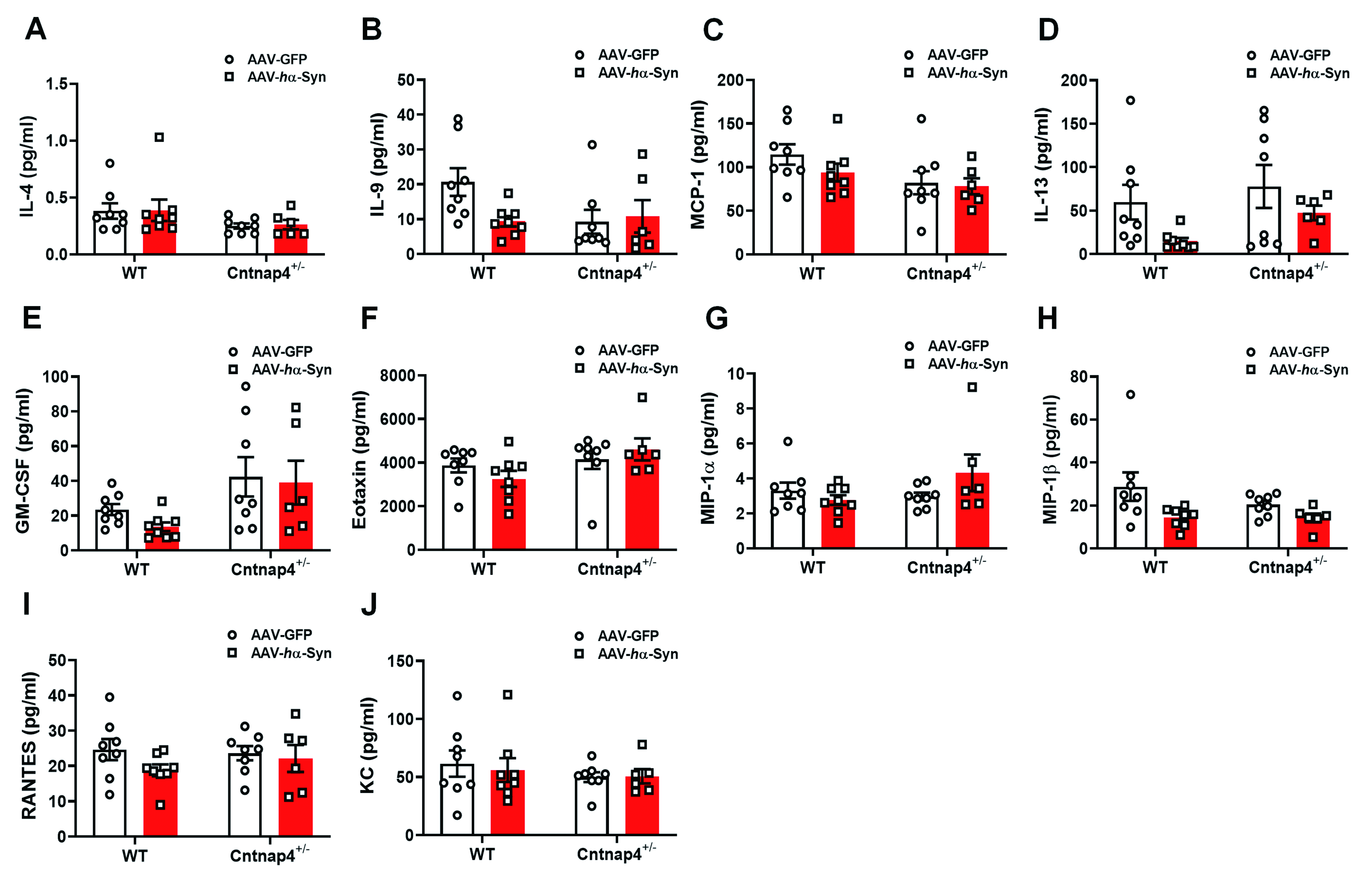

Supplement: Supplementary file 9 — Supplementary Figure 7 [file 41419_2023_5807_MOESM9_ESM.tif]

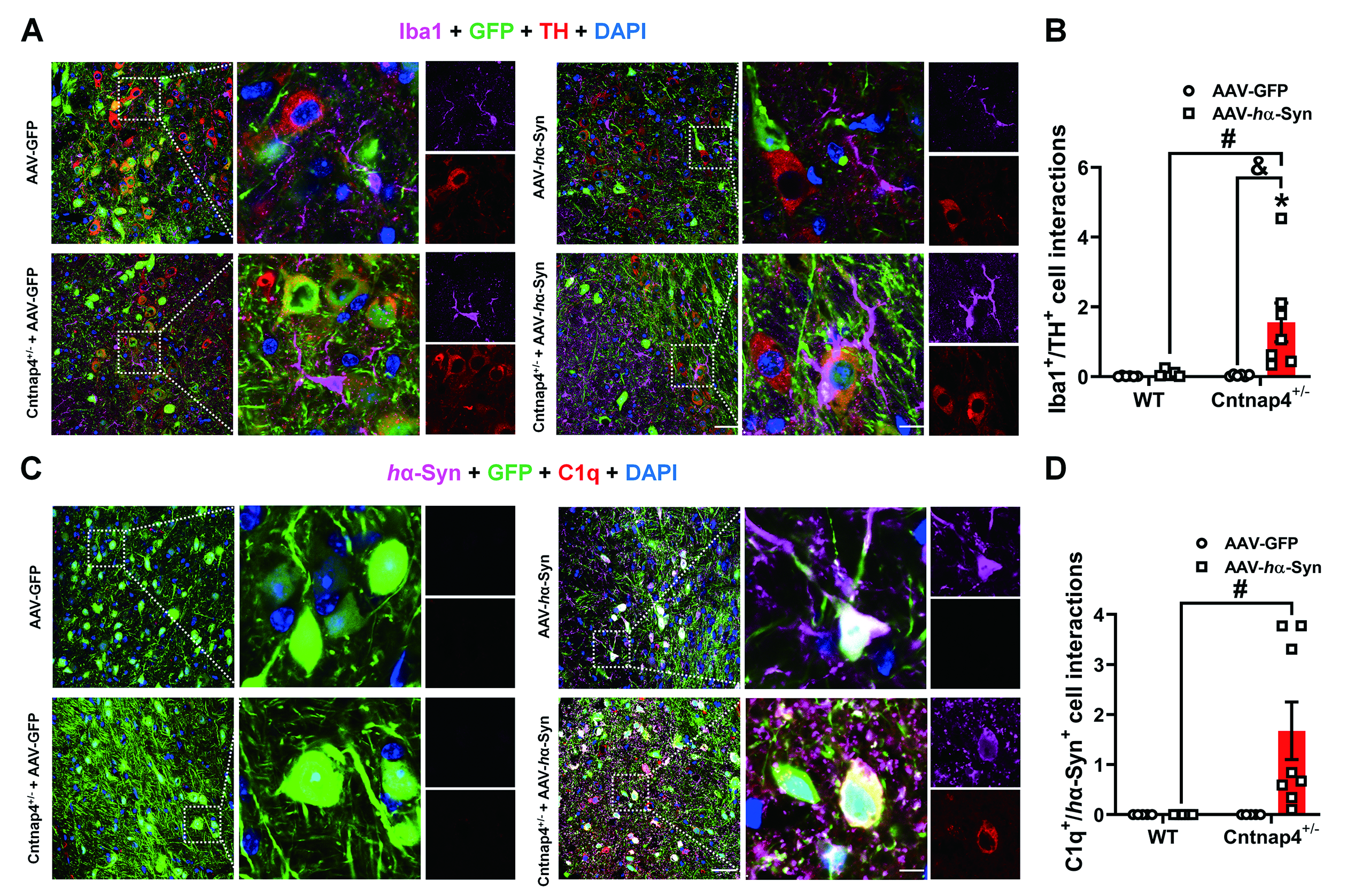

Supplement: Supplementary file 10 — Supplementary Figure 8 [file 41419_2023_5807_MOESM10_ESM.tif]

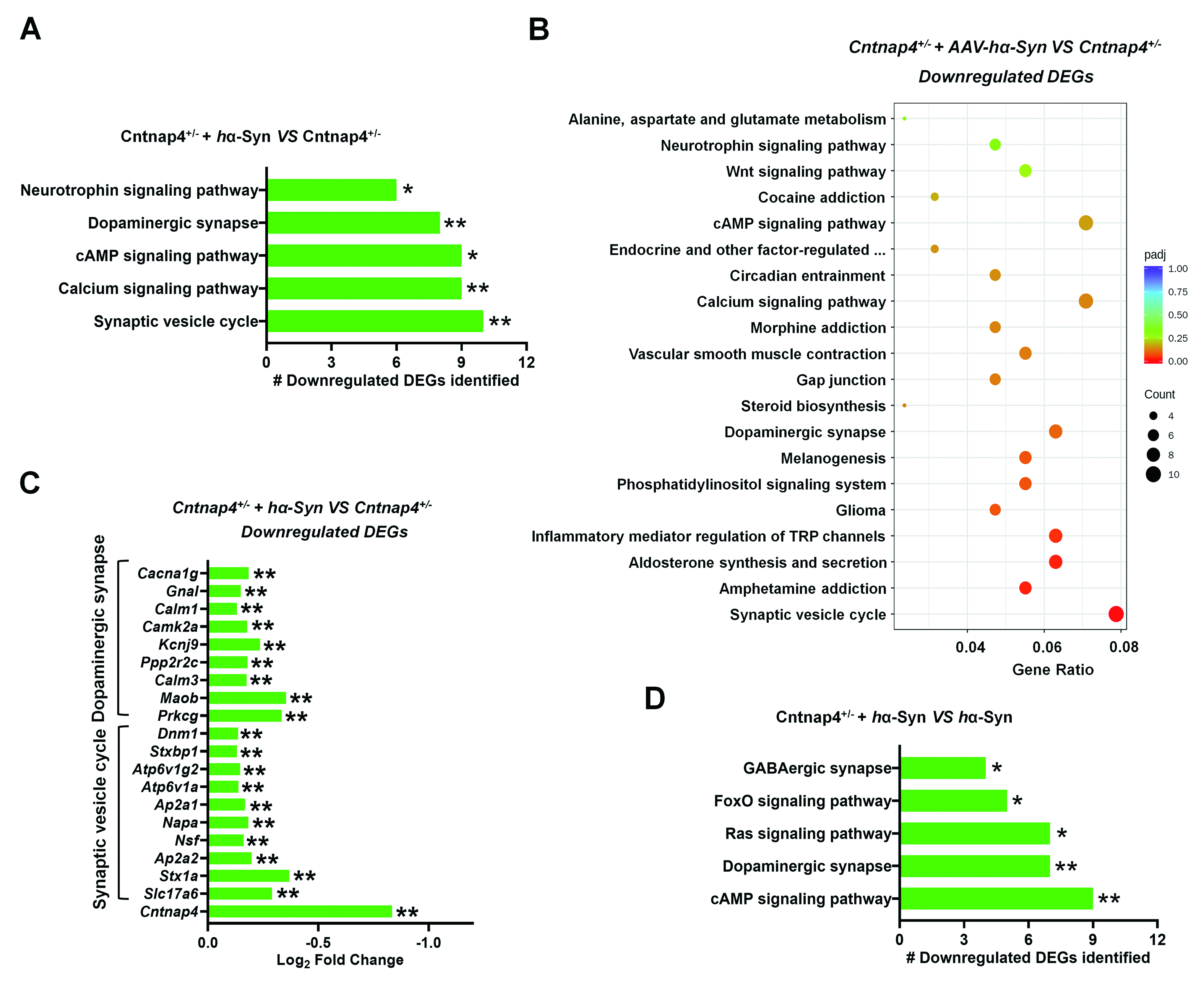

Supplement: Supplementary file 11 — Supplementary Figure 9 [file 41419_2023_5807_MOESM11_ESM.tif]

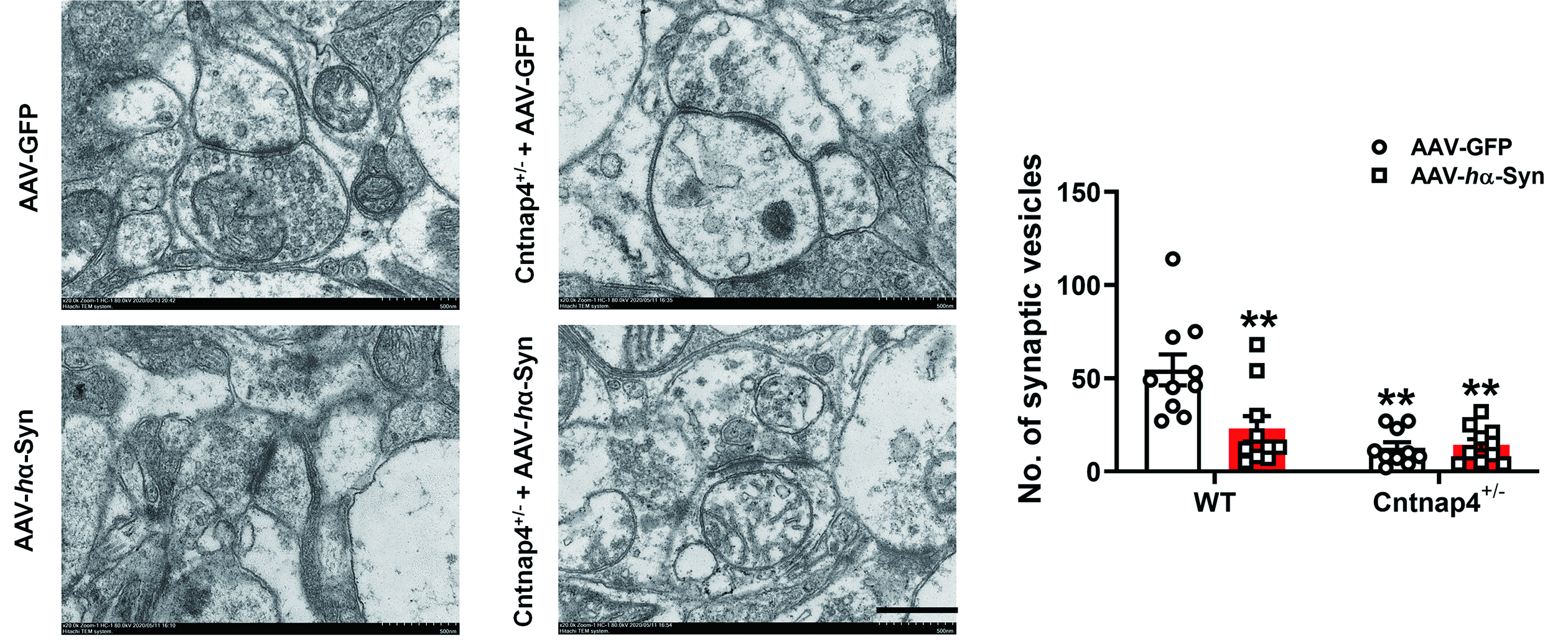

Supplement: Supplementary file 12 — Supplementary Figure 10 [file 41419_2023_5807_MOESM12_ESM.tif]

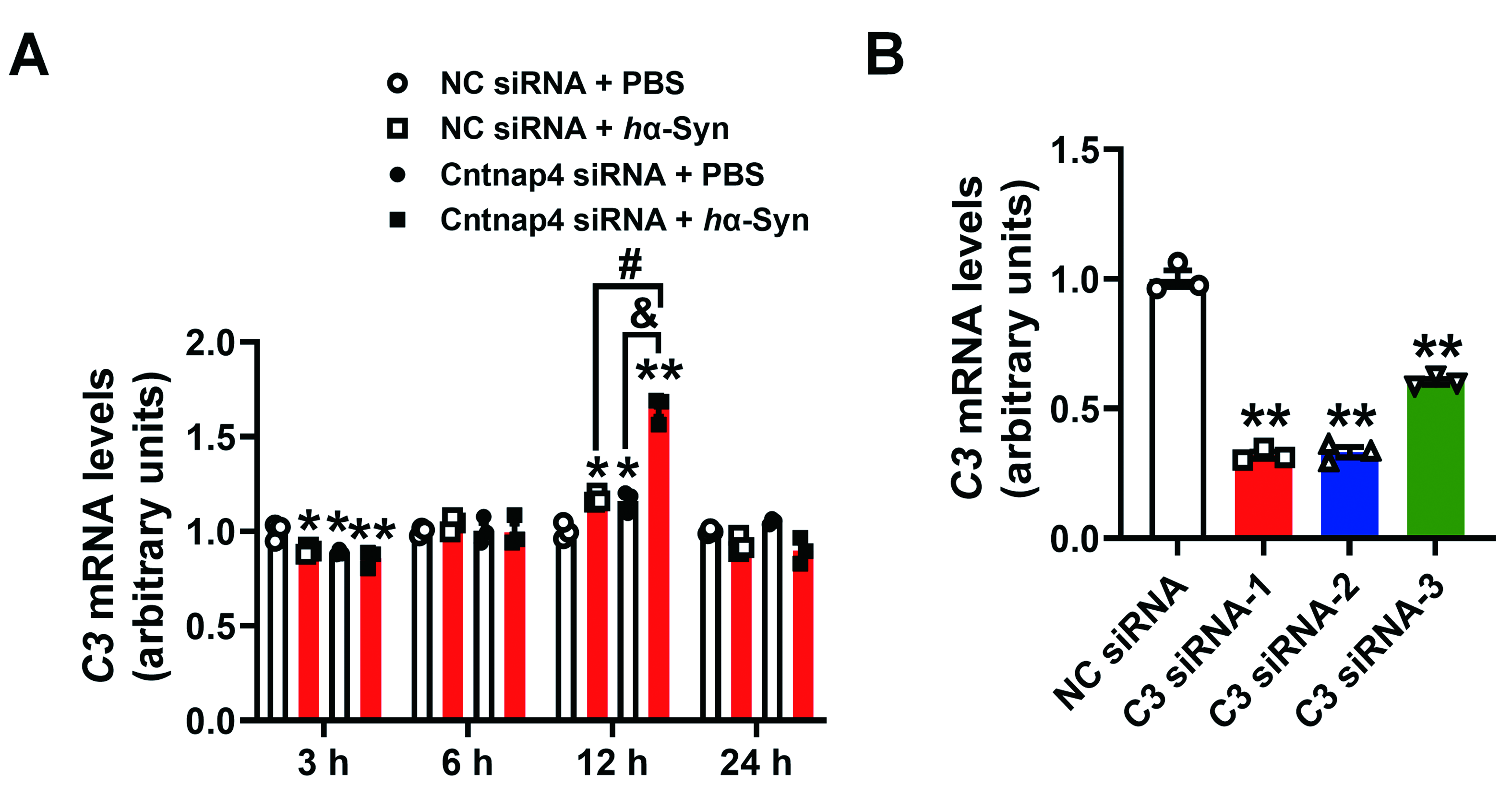

Supplement: Supplementary file 13 — Supplementary Figure 11 [file 41419_2023_5807_MOESM13_ESM.tif]

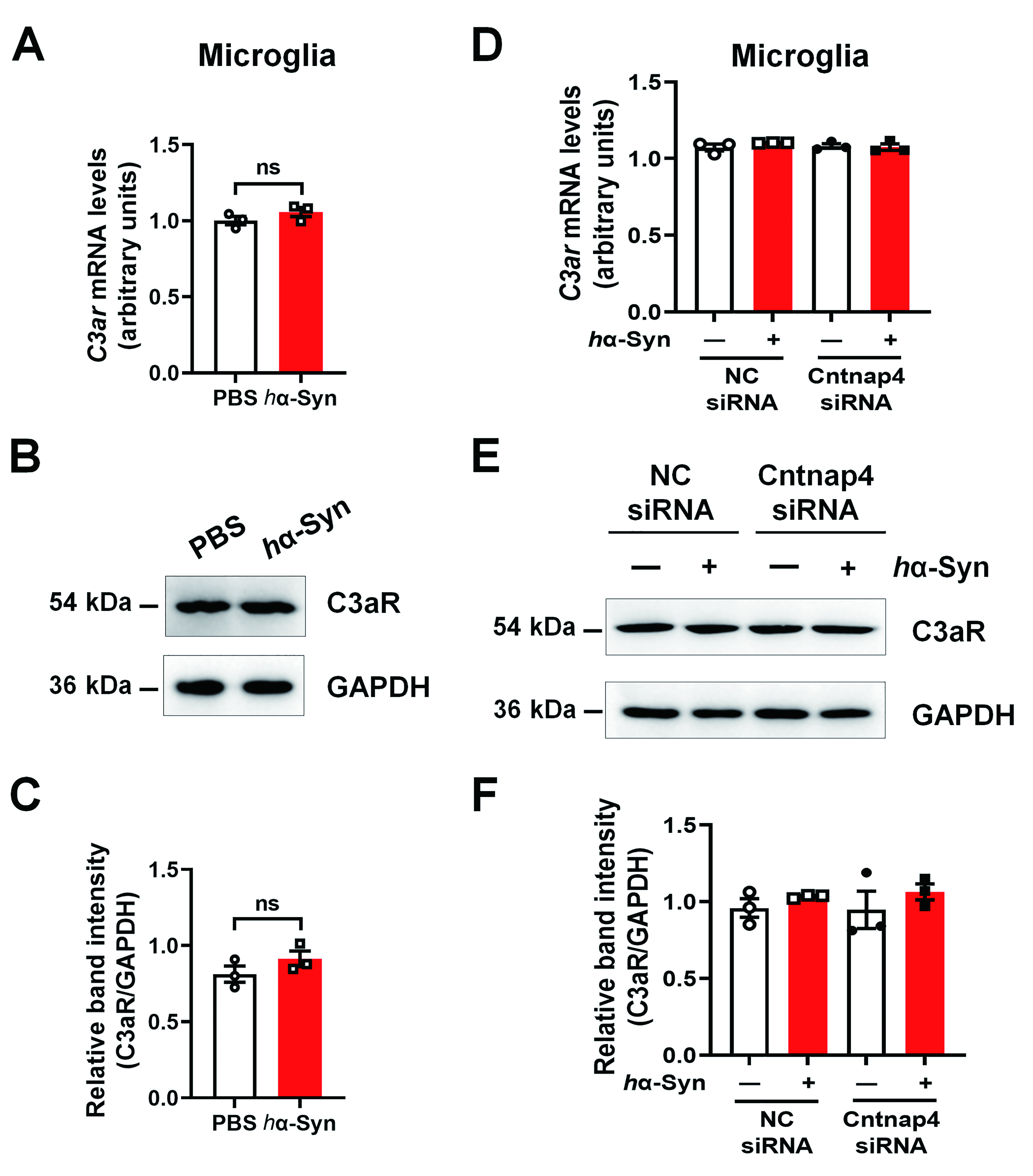

Supplement: Supplementary file 14 — Supplementary Figure 12 [file 41419_2023_5807_MOESM14_ESM.tif]

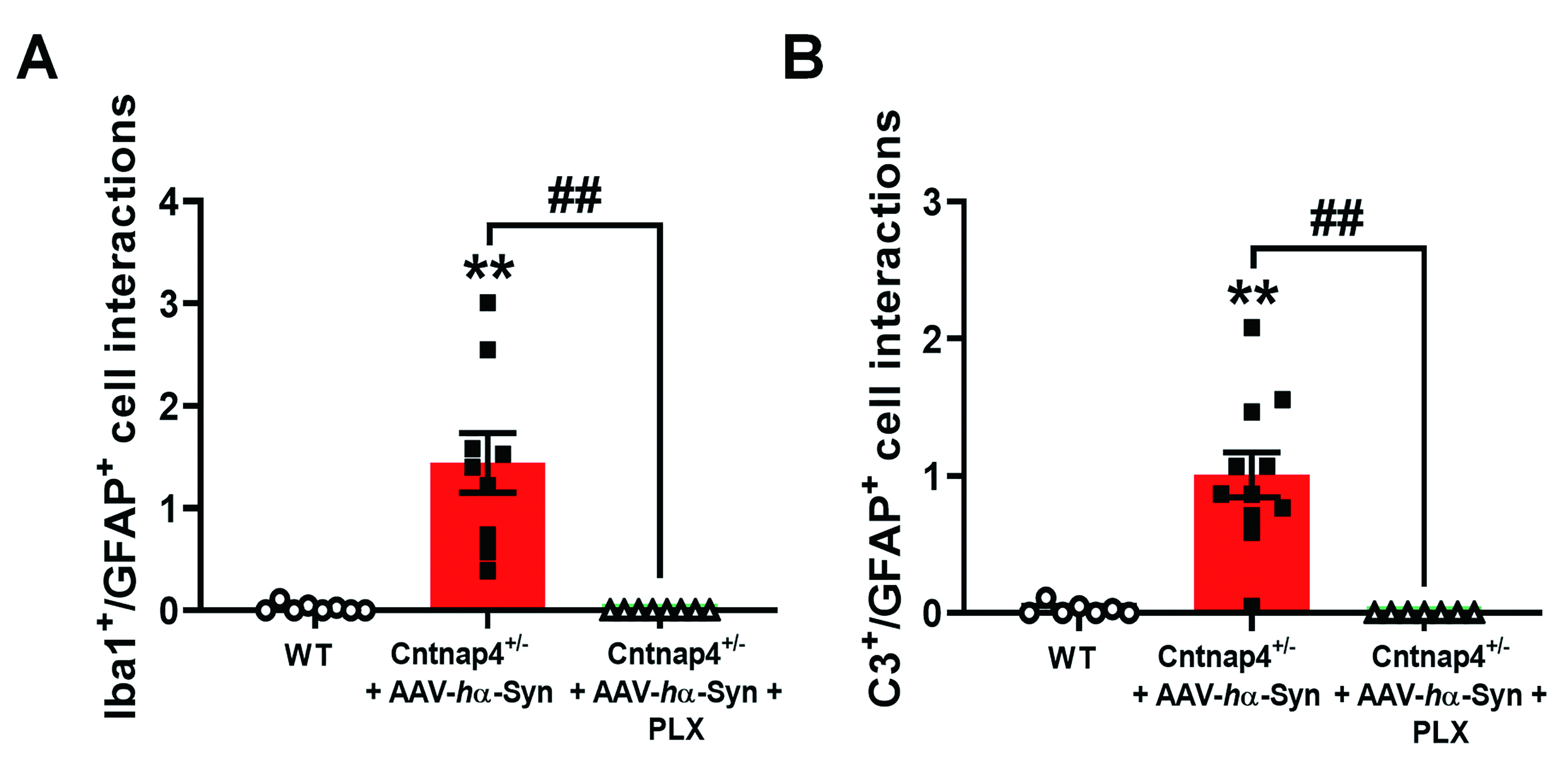

Supplement: Supplementary file 15 — Supplementary Figure 13 [file 41419_2023_5807_MOESM15_ESM.tif]

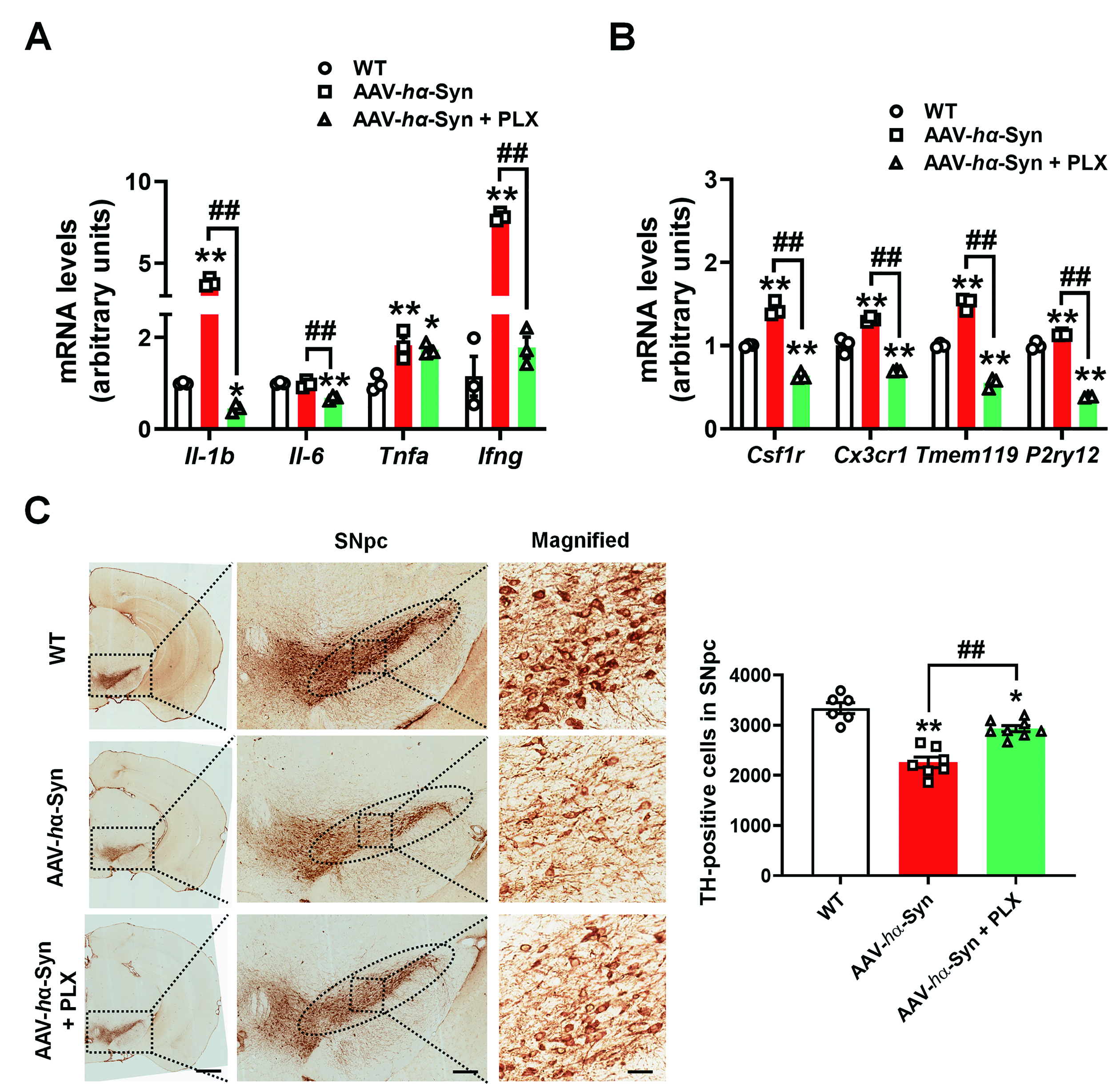

Supplement: Supplementary file 16 — Supplementary Figure 14 [file 41419_2023_5807_MOESM16_ESM.tif]

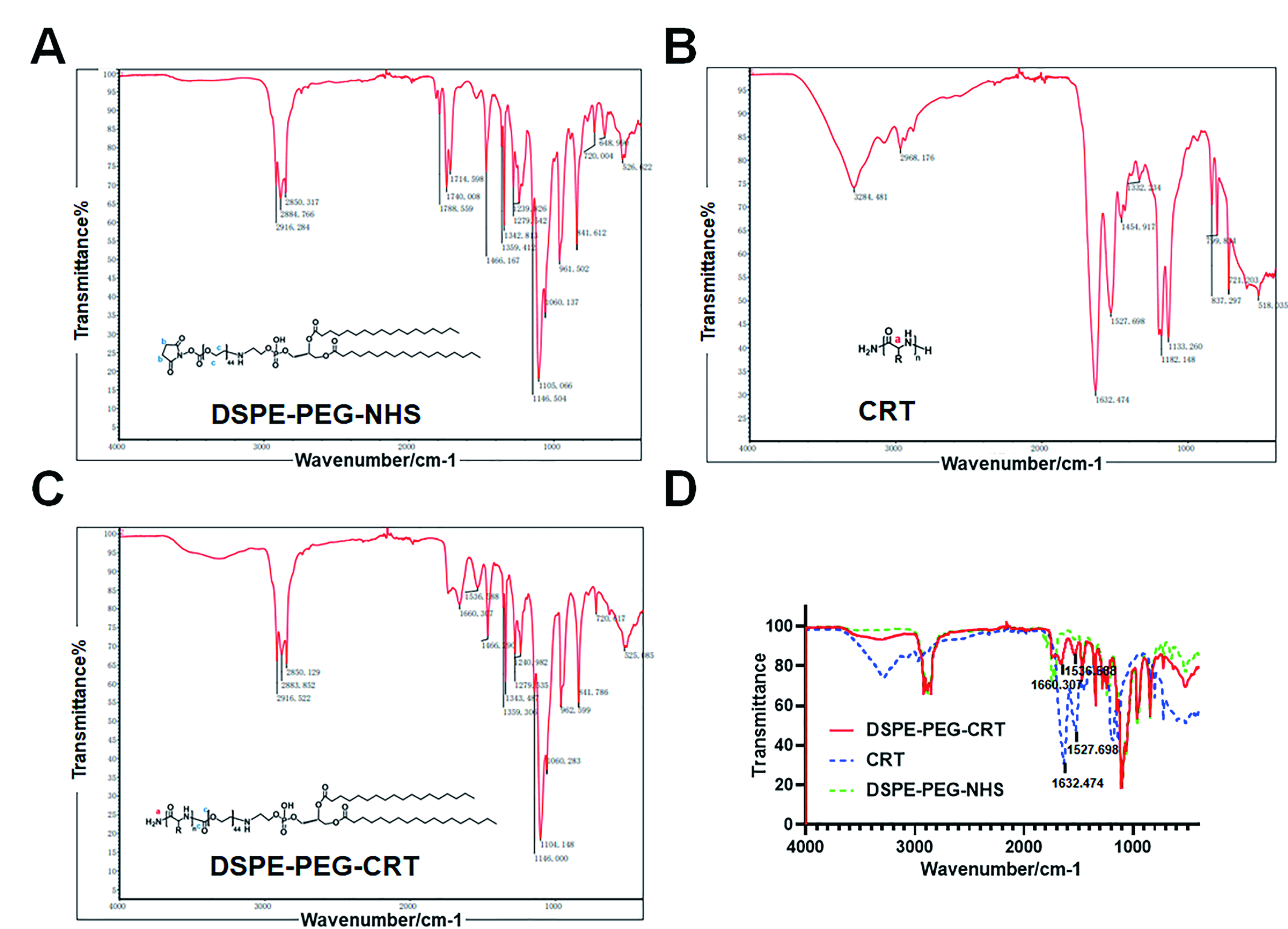

Supplement: Supplementary file 17 — Supplementary Figure 15 [file 41419_2023_5807_MOESM17_ESM.tif]

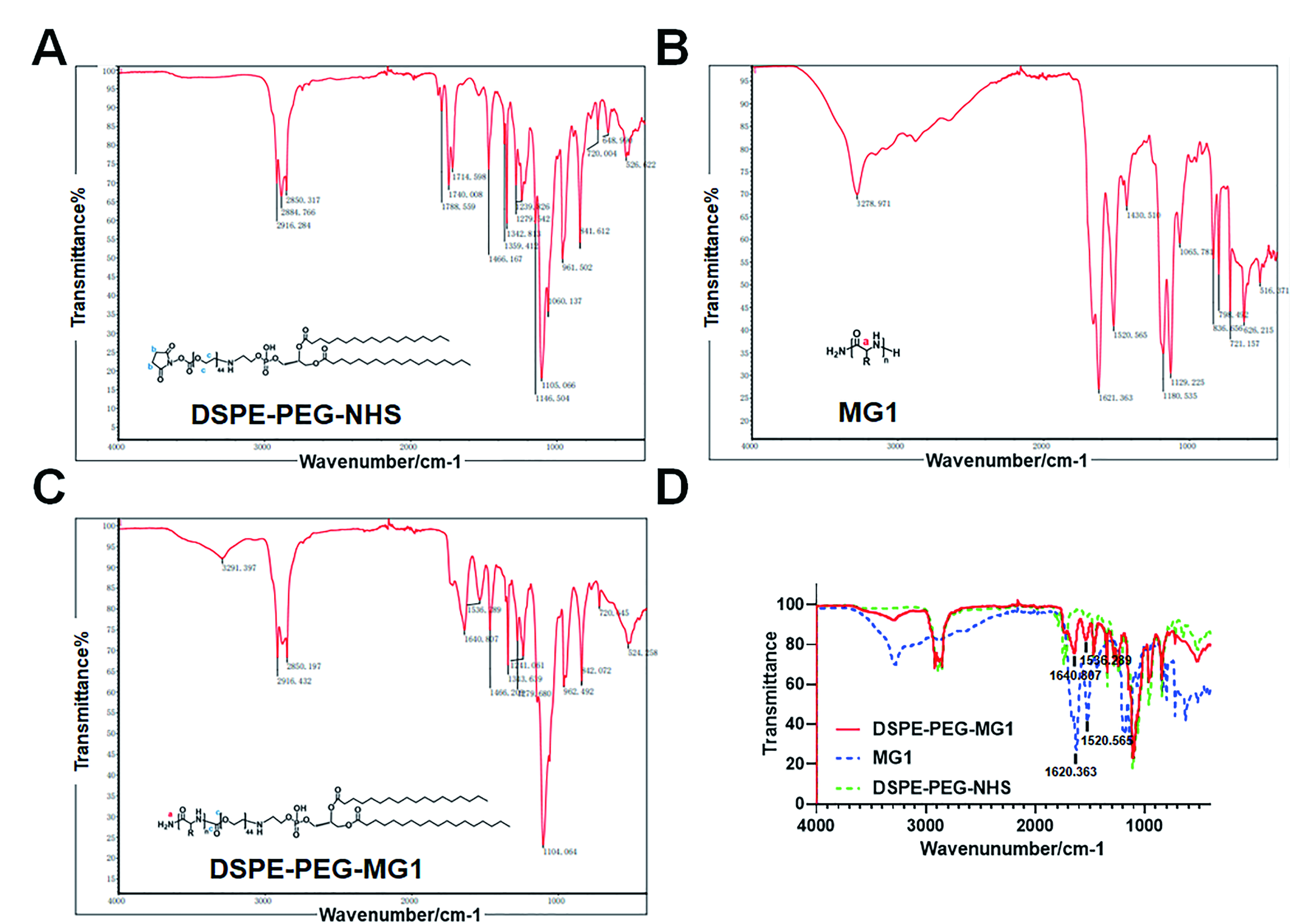

Supplement: Supplementary file 18 — Supplementary Figure 16 [file 41419_2023_5807_MOESM18_ESM.tif]

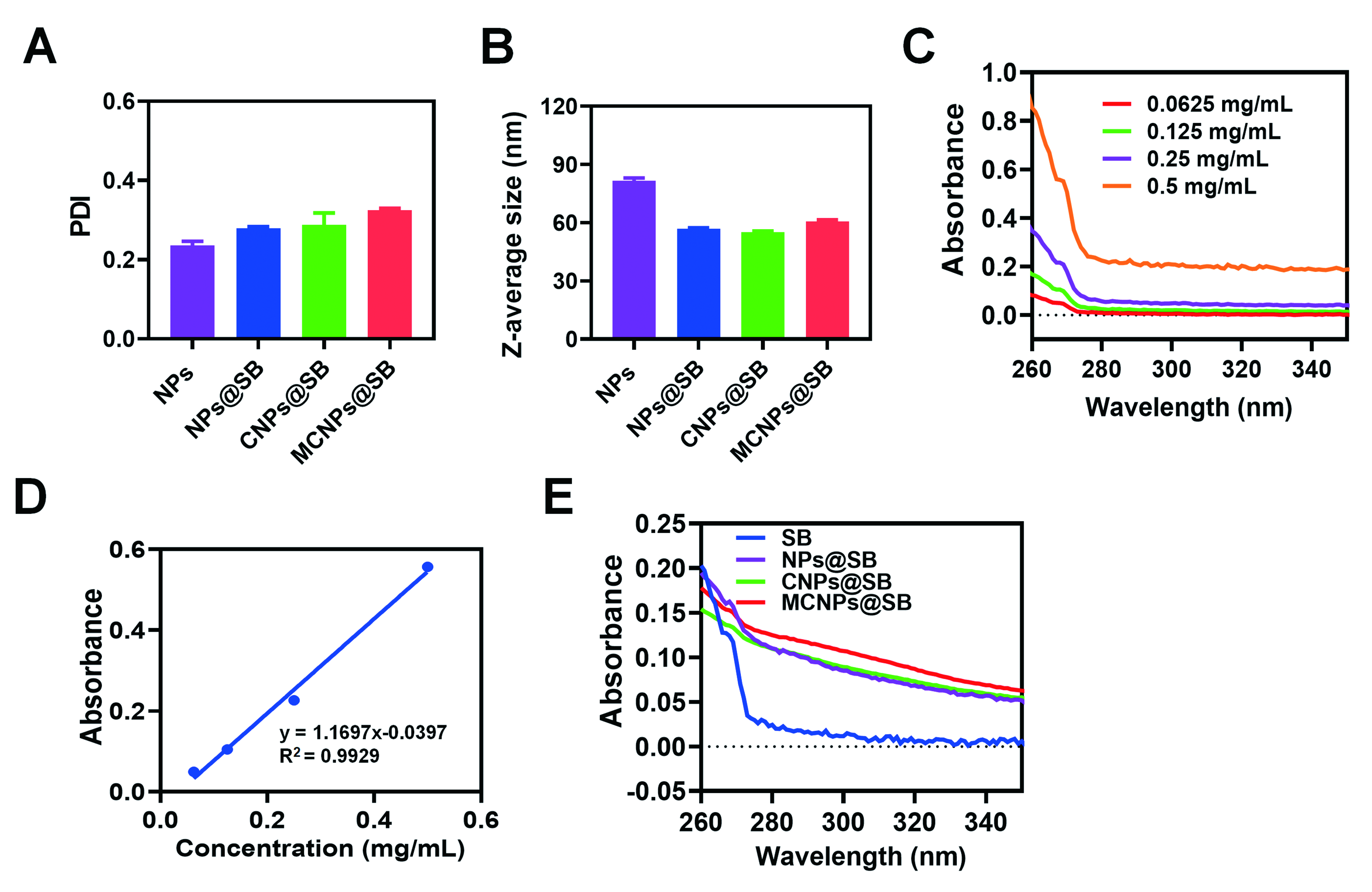

Supplement: Supplementary file 19 — Supplementary Figure 17 [file 41419_2023_5807_MOESM19_ESM.tif]

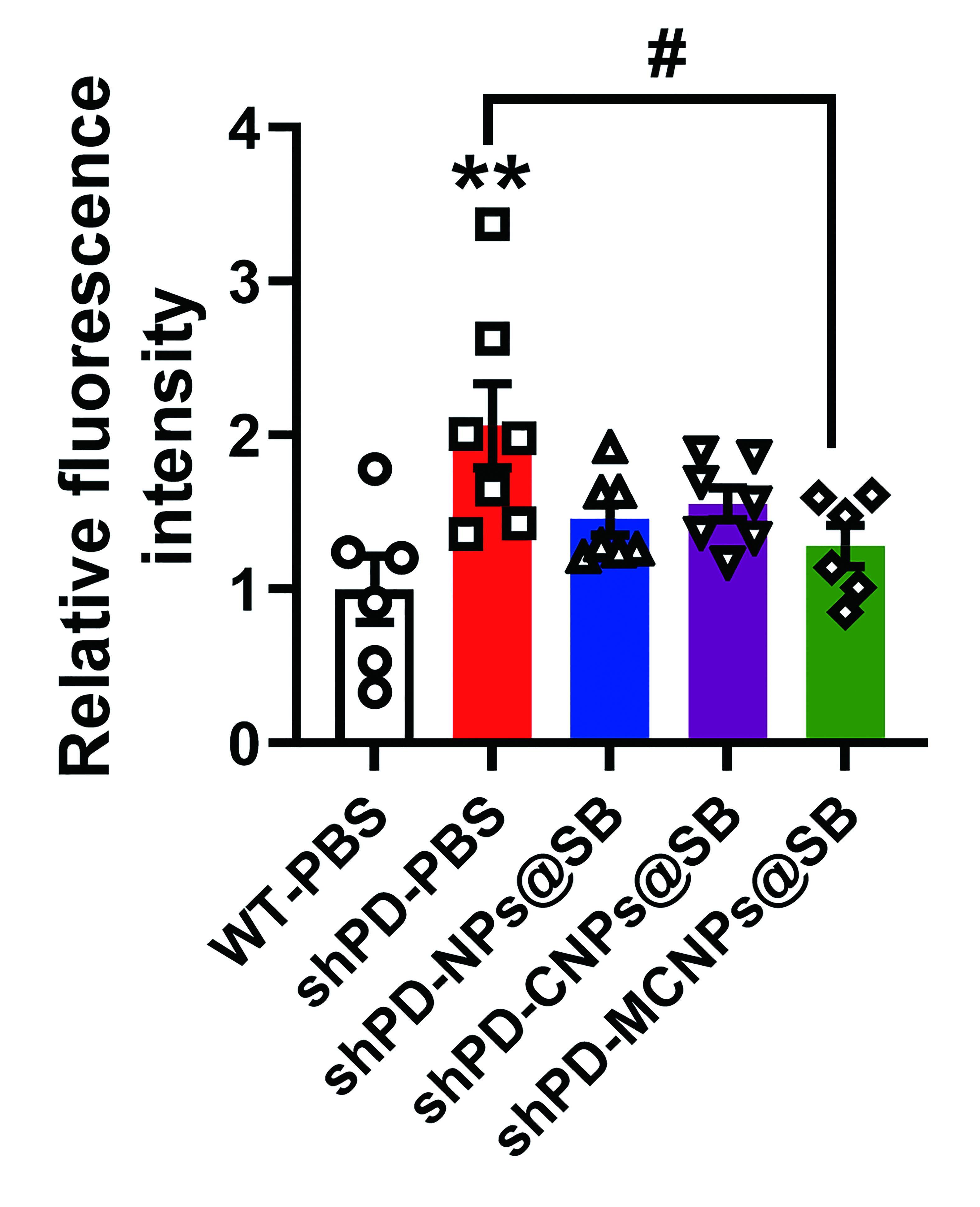

Supplement: Supplementary file 20 — Supplementary Figure 18 [file 41419_2023_5807_MOESM20_ESM.tif]
