# Supplementary material for: Cntnap4 partial deficiency exacerbates α-synuclein pathology through astrocyte–microglia C3-C3aR pathway
Source: Cell Death Dis. 2023 Apr 22;14(4):285. doi: 10.1038/s41419-023-05807-y (PMC10122675; doi:10.1038/s41419-023-05807-y)

Figure 1H

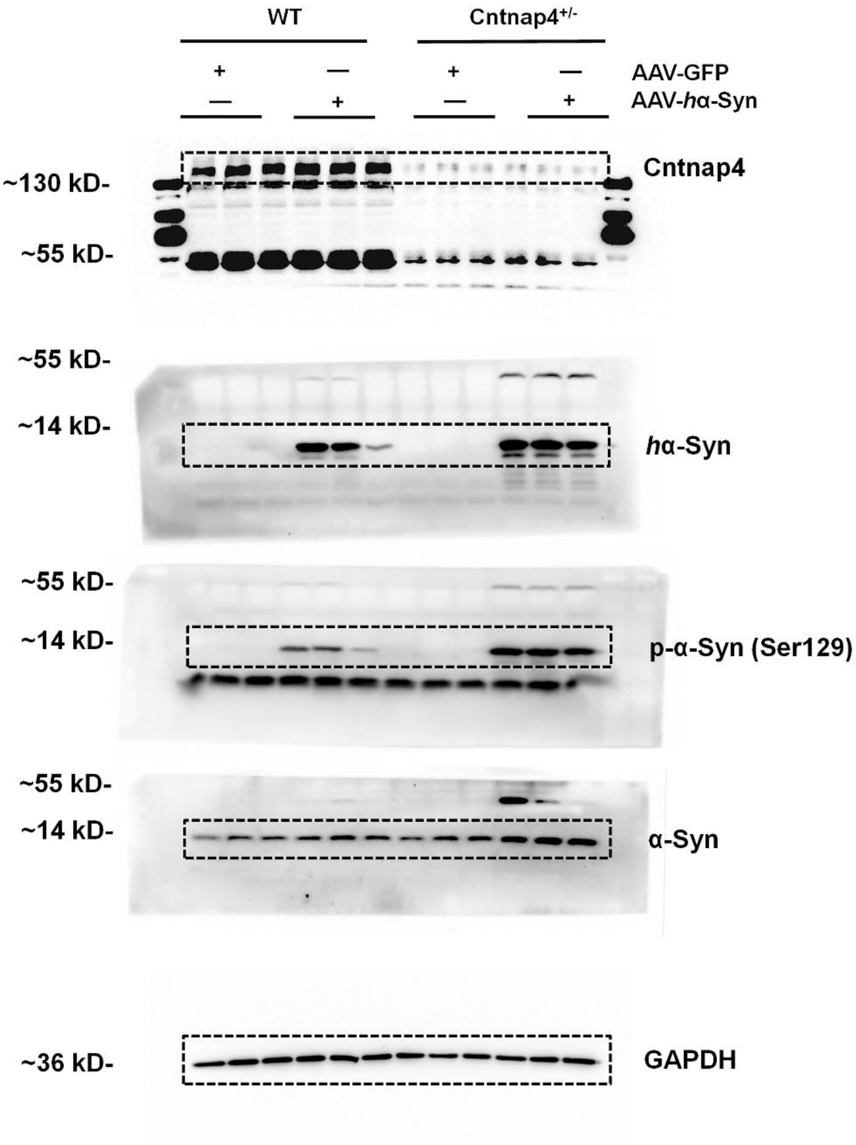
WT
Cntnap4+/-
AAV-GFP
AAV-hα-Syn
~130 kD-
~55 kD-
Cntnap4
~55 kD-
~14 kD-
hα-Syn
~55 kD-
~14 kD-
p-α-Syn (Ser129)
~55 kD-
~14 kD-
α-Syn
~36 kD-
GAPDH

Figure 2D

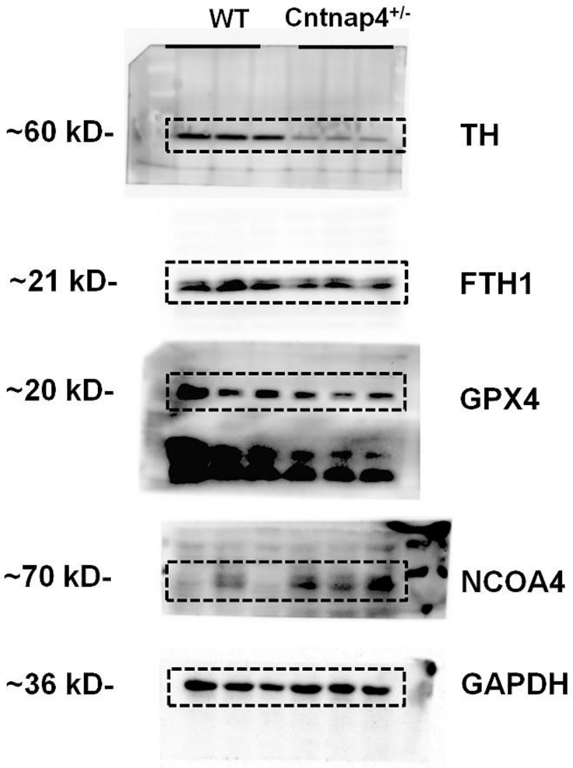

Figure 2F

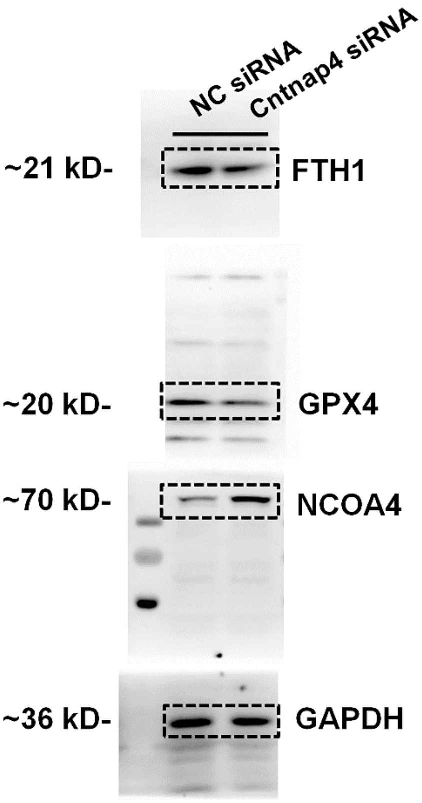

Figure 2N

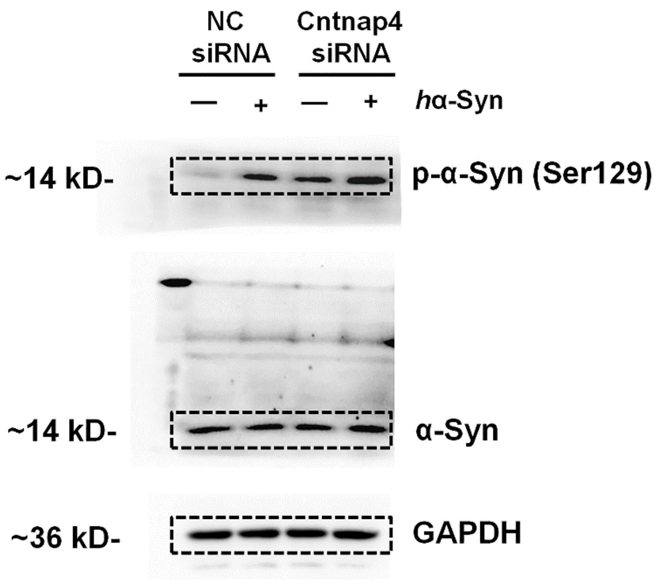

Figure 2P

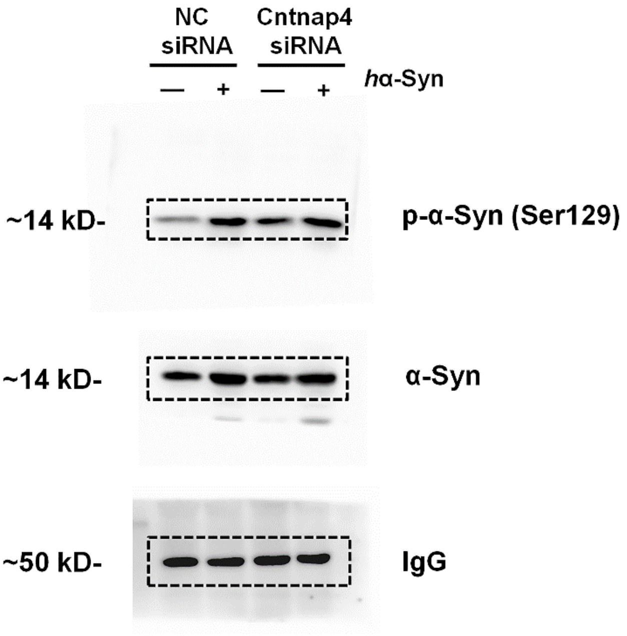

Figure 4K

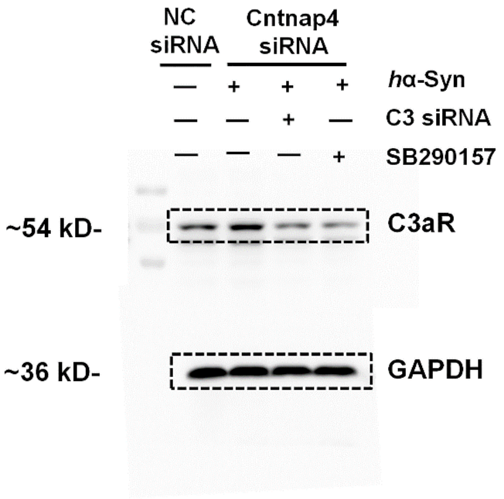

Figure 5Q

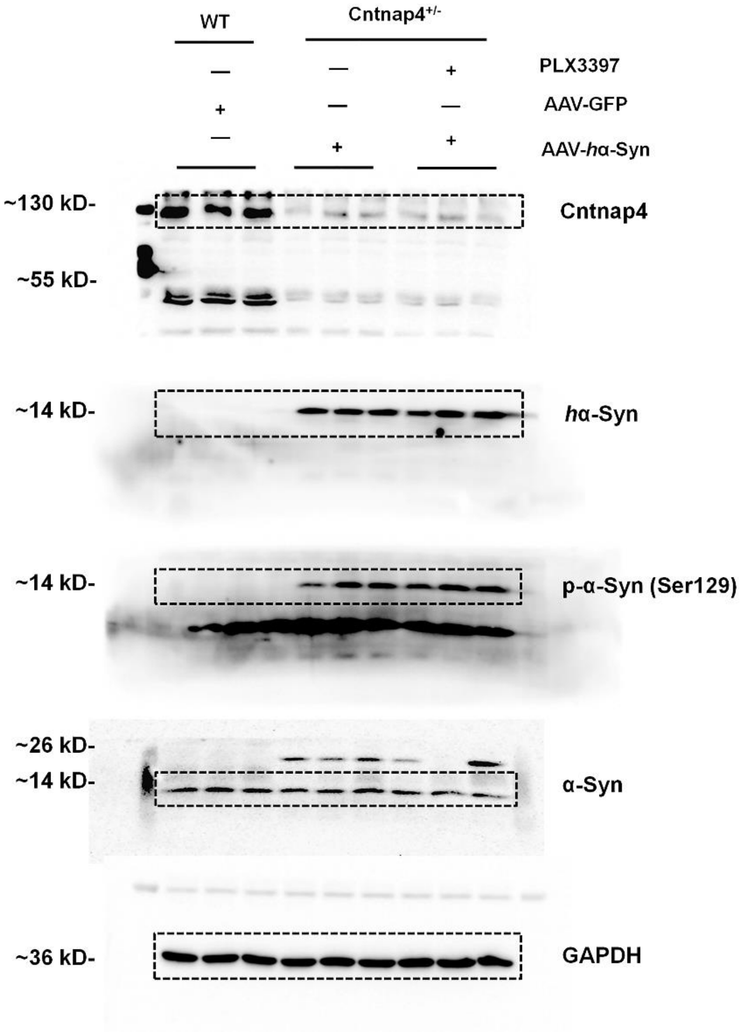

Figure 6F

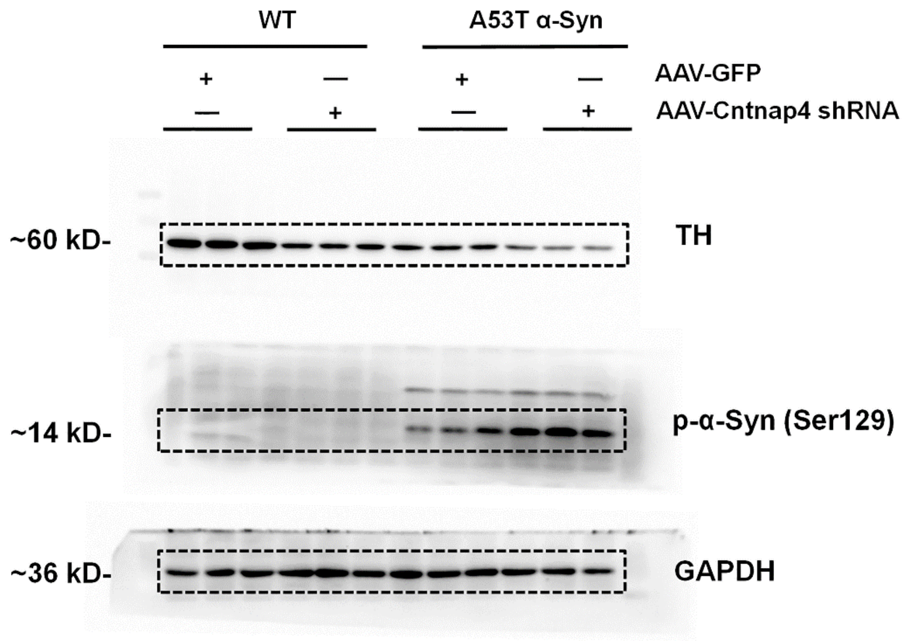

Figure 8F

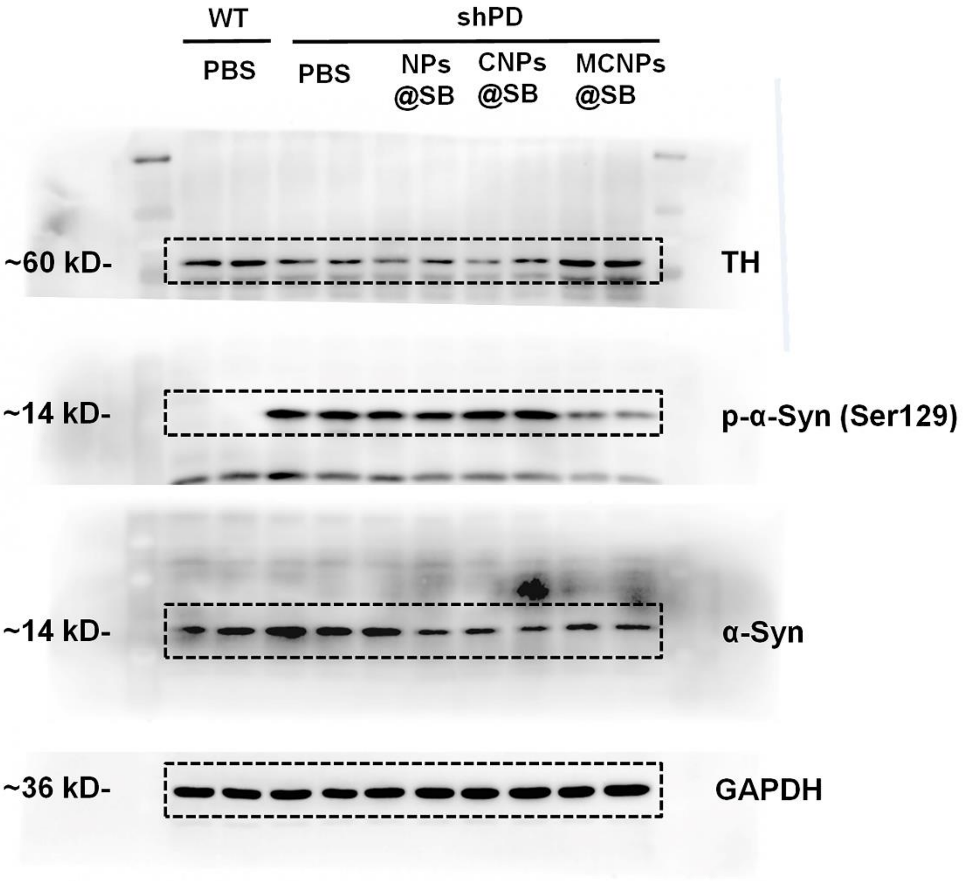

Figure S1C

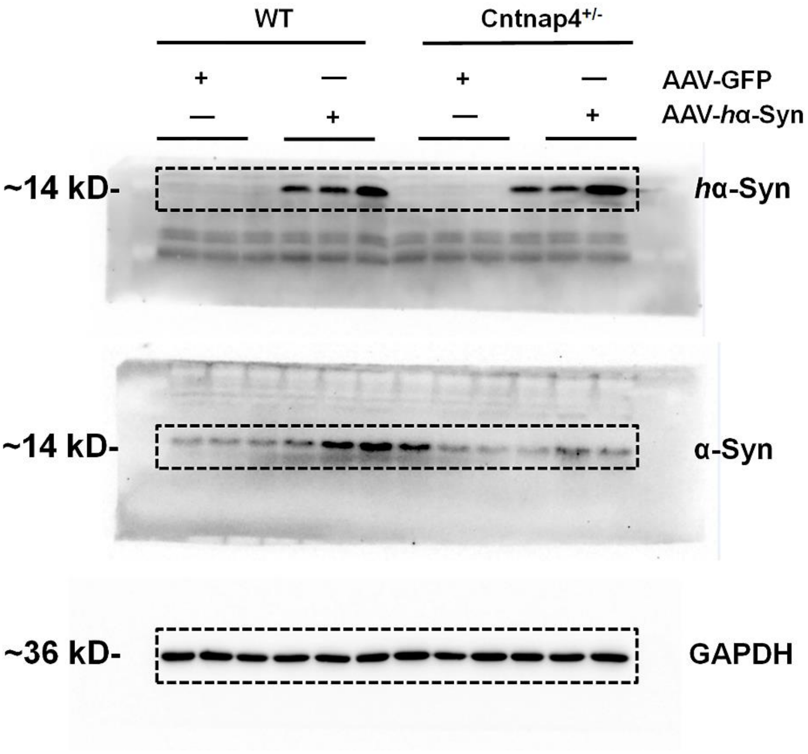

Figure S2A

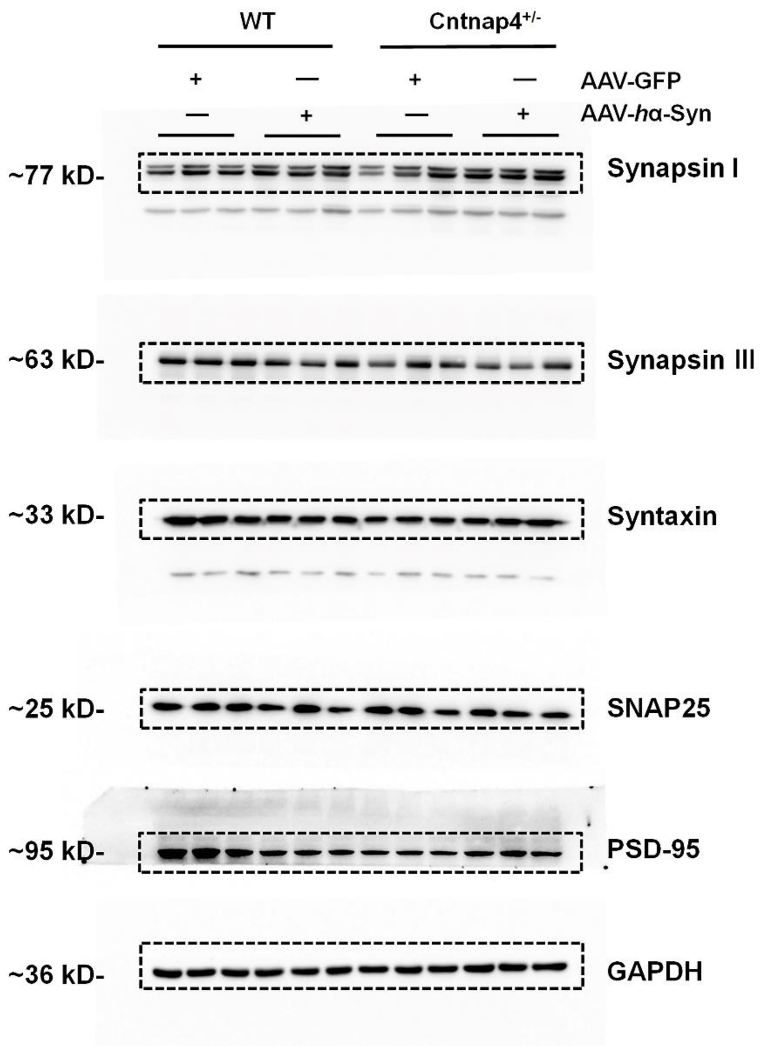
WT
Cntnap4+/-
AAV-GFP
AAV-hα-Syn
~77 kD-
Synapsin I
~63 kD-
Synapsin III
~33 kD-
Syntaxin
~25 kD-
SNAP25
~95 kD-
PSD-95
~36 kD-
GAPDH

Figure S12B

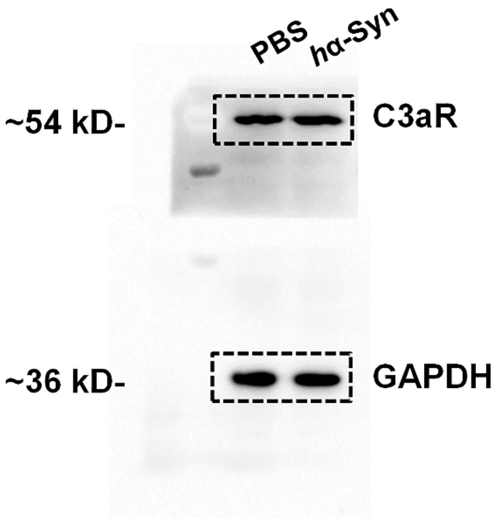

Figure S12E

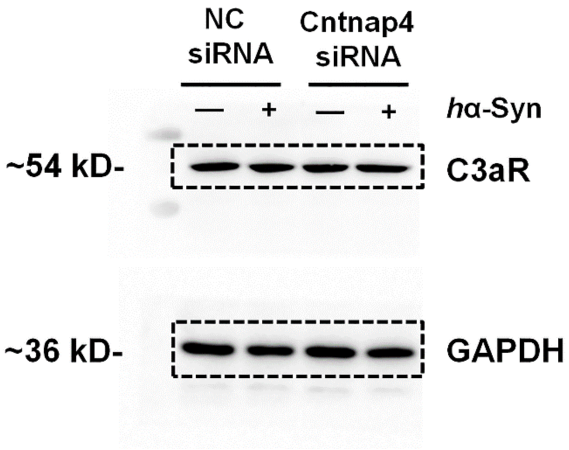

Supplement: Supplementary file 22 — Raw images for western blots [file 41419_2023_5807_MOESM22_ESM.pdf]
